# Integrating bioactivity and molecular simulations to explore the pharmacological landscape of *Lagerstroemia speciosa* leaf extract

**DOI:** 10.1371/journal.pone.0339566

**Published:** 2025-12-29

**Authors:** Md Shajedul Haque, Rubel Ahmad, Bidhan Chandra Sarkar, Manoshi Sana, Hasi Rani Saha, Niranjan Kumar Sana

**Affiliations:** 1 Department of Biochemistry and Molecular Biology, University of Rajshahi, Rajshahi, Bangladesh; 2 Department of Biochemistry and Molecular Biology, Primeasia University, Dhaka, Bangladesh; 3 Dhaka Medical College Hospital, Dhaka, Bangladesh; Universidade Federal do Para, BRAZIL

## Abstract

Natural resources are vital for identifying novel treatments for noncommunicable diseases and multidrug-resistant pathogens. *Lagerstroemia speciosa (L. speciosa)* Linn. is traditionally used to manage conditions like diabetes, cancer, and oxidative stress-related diseases. Current studies frequently lack comprehensive bioactivity assessments coupled with molecular simulation analyses of *L. speciosa* leaf extracts. We aimed to assess the phytochemical profiling, bioactivity, *in silico* pharmacological properties, and molecular interactions of *L. speciosa* leaf extracts prepared using organic solvents. Methanol (5.1) and ethanol (4.3) extracts yielded the highest concentrations of total phenolics, flavonoids, and proanthocyanidins due to their high polarity indices. Antioxidant activities were robustly evaluated using DPPH, ABTS, superoxide, and nitric oxide assays, with the ethanol extract demonstrating significant free radical scavenging (IC_50_ = 75.53 µg/mL for DPPH). The methanol extract exhibited broad-spectrum antibacterial activity against multidrug-resistant strains, including *Escherichia coli* and *Staphylococcus aureus*, alongside moderate antifungal activity against *Candida albicans* and *Saccharomyces cerevisiae*. The brine shrimp lethality assay revealed moderate cytotoxicity (LC_50_ = 601.8 µg/mL), suggesting potential anticancer properties likely mediated by bioactive compounds such as ellagic acid and gallic acid. Methanol and ethanol extracts of *L. speciosa* significantly inhibited α-amylase and α-glucosidase compared to standard acarbose, indicating substantial antidiabetic potential by delaying carbohydrate digestion and reducing postprandial glucose levels. Complementary molecular docking and ADME pharmacokinetic studies provided *in silico* support for the observed antioxidant, antimicrobial, antidiabetic, and chemopreventive activities. The compelling evidence collectively suggests that *L. speciosa* extracts are a promising source of bioactive compounds.

## Introduction

Noncommunicable diseases are increasingly becoming major global health concerns, with over two billion people affected by conditions linked to oxidative stress [1–3]. Oxidative stress can be mitigated by bioactive compounds with antioxidant properties present in the body [4]. These compounds enhance the production of endogenous antioxidants, including SOD (superoxide dismutase), GPx (glutathione peroxidase), and catalase. These enzymes are essential for protecting the vascular endothelium against free radical-induced damage [5]. Antioxidants are necessary compounds that play a vital role in maintaining human health [6]. The plant kingdom represents a rich source of potential antioxidants for combating oxidative stress-related diseases [7]. Antimicrobial resistance is approaching critical levels, with global deaths projected to reach ten million by the 2030s [8,9]. This evolving health crisis emphasizes the urgent need for novel therapeutic agents derived from natural sources [10,11]. Natural products are inherently more complex, functioning as multitarget molecules. This complexity often leads to better therapeutic outcomes and fewer side effects compared to synthetic molecules. Synthetic molecules typically target a single receptor site [12,13]. Reactive oxygen species (ROS) are continuously generated in human cells. These include superoxide radicals, hydroxyl radicals, singlet oxygen, hydrogen peroxide, and nitric oxide [14]. These reactive entities damage cellular components such as membrane lipids, proteins, enzymes, and DNA [15]. As a result, natural products have gained significant scientific interest for their antioxidant and chemo-preventive properties [16].

*L. speciosa* Linn. is commonly known as ‘Banaba’ in the Tagalog dialect of the Philippines [17]. In Bangladesh, it is locally referred to as ‘Jarul’. This tropical flowering tree belongs to the family Lythraceae. It is a medium-to-large deciduous tree distinguished by its rounded crown. The species is indigenous to Southeast Asia, with its native range encompassing Bangladesh, India, Indonesia, the Philippines, and Malaysia [18]. The plant has been widely used in traditional medicine across these regions to treat various ailments [19]. Banaba has been used as a traditional diabetes remedy in the Philippines since ancient times [20]. In Malaysian traditional medicine, the bark of *L. speciosa* has been used as a treatment for dysentery and diarrhea [21]. The study on Banaba indicated that pure corosolic acid significantly reduced blood glucose levels in humans [22,23]. It exhibits diverse pharmacological effects, including anti-inflammatory, analgesic, hepatoprotective, nephroprotective, anti-gout and alpha-amylase inhibitory properties [24–27]. These findings highlight its potential as a versatile therapeutic agent.

Previous studies have demonstrated the antioxidant capabilities and α-glucosidase inhibitory activity of its leaf extracts [28]. However, comprehensive investigations using diverse assays and exploring multiple mechanistic pathways are still lacking. The effects of *L. speciosa* on other metabolically critical enzymes, such as α-amylase, lipase, and tyrosinase, have not been systematically investigated [28]. This limitation hinders the comprehensive utilization of its therapeutic potential. Toxicological evaluations have largely been confined to acute toxicity assessments [29,30]. Chronic toxicity, genotoxicity, and organ-specific toxicity profiles remain underexplored. Moreover, safety parameters for various extraction methods and their associated bioactive compounds are to be clearly defined yet [19]. Current literature notably lacks robust structure-activity relationship studies and comprehensive molecular simulations. These relationships are essential to elucidate the connection between phytochemicals and their biological functions. Although numerous bioactive compounds have been identified, their synergistic interactions are not fully understood yet. Addressing these critical gaps is essential to unlock the full medicinal potential of *L. speciosa*.

This study systematically evaluates the bioactive potential of *L. speciosa* leaf extracts obtained through sequential solvent extraction using methanol, ethanol, acetone, and n-hexane. A multiparametric assessment of antioxidant, antimicrobial, cytotoxic and enzymatic inhibition properties was conducted to determine the extracts’ safety profile and therapeutic index for drug development potential. We further employed comprehensive phytochemical profiling, pharmacokinetics, and molecular docking to quantify and characterize the bioactive compounds present in *L. speciosa* leaf extracts. Our research represents a major advancement in the field of phytochemistry by establishing structure-activity connections that bridge the gap between classical ethnopharmacology and contemporary drug discovery.

## Materials and methods

### Chemicals and reagents

All chemicals used were of analytical grade. Folin-Ciocalteu reagent (FCR), 2,2-diphenyl-1-picryl-hydrazyl (DPPH), ABTS [2,2′-azino-bis (3-ethylbenzothiazoline-6-sulfonic acid) diammonium salt], 3,5-dinitrosalicylic acid (DNSA), Dimethyl sulfoxide (DMSO), Griess reagent (1% sulfanilamide, 2% H_3_PO_4_, and 0.1% naphthyl ethylenediamine dihydrochloride), PBS, NaCl, methanol, and catechin (CA) were purchased from Sigma Aldrich, MO, USA. Other reagents for the extraction, such as n-hexane, acetone, ethanol, peptone, agar, nystatin, α-glucosidase, α-amylase, vanillin, and ampicillin, were purchased from Wako Pure Chemical Industries Ltd., Osaka, Japan, while sodium nitroprusside, HCl, sodium acetate, and gallic acid were obtained from Tokyo Chemical Industry Co., Ltd., Tokyo, Japan. Aluminum chloride (AlCl_3_), sodium carbonate, Nitro blue tetrazolium (NBT), sodium carbonate, trypan blue dye, and other remaining analytical-grade chemicals were purchased from Merck Chemical Company, Darmstadt, Germany.

### Plant material

The leaves of *L. speciosa* used in the present study were sourced from the local gardens of Rajshahi district (24° 22’ 23.911’‘ N and 88° 36’ 17.538 E), Bangladesh, and were harvested in July. Taxonomic authentication of plants was done by Professor Dr. A.H.M. Mahbubur Rahman of the Department of Botany, University of Rajshahi, Bangladesh. A voucher specimen (Accession No. RHB-10) has also been deposited in the University of Rajshahi Herbarium (RUH) according to herbarium codes. Sample collection and preservation were done based on the institutional protocols and policies and national rules governing plant tissue collection.

### Preparation of extracts

The collected leaves were washed with distilled water and air-dried at room temperature in a shaded area for two weeks. The samples were then dried, and the dried leaves were ground into a fine powder using a mechanical grinder and finally placed in a tight container. Around 500 g of dried leaf powder was placed in a clean, flat-bottomed glass container and immersed in 1000 mL of methanol for 15 days at room temperature. The resulting extract was filtered through Whatman No. 1 filter paper and concentrated using a rotary evaporator under reduced pressure at 40°C. We evaporated the total filtrate to dryness in a vacuum at 40°C to yield a brownish-red methanolic extract (350 g). The last extract was transferred to a sealed glass vessel for performing the experiment. We also kept aside the 50 g crude methanolic extract of *L. speciosa* leaves for further experiments. In a similar manner, we distilled other solvents- ethanol, acetone, and n-hexane for extraction from the crude *L. speciosa* leaves for the experiments in this study.

### Total phenolic content

The total phenolic content (TPC) of the *L. speciosa* leaf extracts was estimated using the Folin Ciocalteu reagent (FCR) assay [31]. When 5 mL of FCR was mixed with 1 mL of methanol containing gallic acid, the mixture was then incubated with *L. speciosa* for 5 mins at room temperature. After that, an equal volume of 1 mL of 7.5% sodium carbonate (Na_2_CO_3_) was also added into the mixture. To the above mixture, we added distilled water up to 10 mL and stirred it well. The optical density at 765 nm was measured in a UV-visible spectrophotometer (Shimadzu, Japan) after incubation at room temperature for 60 minutes, with methanol serving as the blank. Similarly, we followed the same protocol to measure the total phenolic content for the extracts obtained using other solvents. Total phenolics of the extract were determined using gallic acid as a standard reference. The values were expressed as milligrams of gallic acid equivalent (GAE) per gram of the dry extract. A calibration curve for gallic acid was constructed using the linear regression equation y = 0.004797*x + 0.04165, demonstrating a strong correlation with a coefficient of determination (R^2^) of 0.9648 (S1 Fig.).

### Total flavonoid content

The total flavonoid content (TFC) of *L. speciosa* leaf extracts was measured using a colorimetric assay by the aluminum chloride (AlCl_3_) method [32]. A mixture was prepared by adding 1 mL of *L. speciosa* leaf extract, 5 mL of distilled water, and 0.3 mL of 5% NaNO_2_ in a test tube and allowed to stand for 5 minutes. Following the incubation period, we added 0.6 mL of 10% aluminum chloride, let it stand for 5 minutes, and then added 2 mL of 1M NaOH. At a wavelength of 510 nm, the absorbance of the reaction mixture was measured. A calibration curve for catechin was established using the linear regression equation y = 0.002137*x + 0.03542, which exhibited a strong linear relationship with a coefficient of determination (R^2^) of 0.9917. The flavonoid content was quantified based on the catechin standard and expressed as milligrams of catechin equivalent (CE) per gram of dry extract (S2 Fig.).

### Total flavonol content

Determination of the total flavonol content was followed by the method described Kumaran et al. [33] with minor changes. Briefly, 2.0 mL of *L. speciosa* leaf extracts at a concentration of 0.1 mg/mL were reacted with 2.0 mL of aluminum chloride at a concentration of 2% w/v in ethanol and 3.0 mL of a sodium acetate solution of 50 g/L. The reaction mixture was then maintained at 20°C for 2.5 hours, after which absorbance was recorded on a UV-visible spectrophotometer (Shimadzu Corporation, Japan) at 440 nm. A calibration curve for quercetin was generated using the linear regression equation y = 0.002490*x + 0.04230, demonstrating a strong linear relationship with a coefficient of determination (R^2^) of 0.9935. The flavonol content was quantified using quercetin as a reference standard and expressed as milligrams of quercetin equivalent (QE) per gram of dry extract (S3 Fig.).

### Total proanthocyanidin content

Total proanthocyanidin content was determined after quantitating using the vanillin-HCl method according to Sun et al. [34] with slight modifications. The reaction mixture for the extract solution consisting of 1.5 mL of extract solution (0.1 mg/mL of methanol) diluted to 3 mL with vanillin reagent (4% w/v in methanol) was first diluted with 1.5 mL of concentrated hydrochloric acid (37% v/v). The chromophores were allowed to develop in the reaction mixture at room temperature for 15 minutes. Absorbance at 500 nm was read on a UV visible spectrophotometer (Shimadzu, Japan). The proanthocyanidin content was determined and expressed as milligrams of catechin equivalents (CE) per gram of dry extract. A standard calibration curve, generated from a series of catechin concentrations, was established using the linear regression equation y = 0.002128*x + 0.01226, demonstrating a robust linear relationship with a coefficient of determination (R^2^) of 0.9949 (S4 Fig).

### DPPH free radical scavenging assay

The *L. speciosa* extracts were employed for their DPPH (2,2-diphenyl-1-picryl-hydrazyl) radical scavenging activity following the methodology of Brand-Williams et al. [35]. We prepared concentrations of 0.5 mg/mL by dissolving 5 mg of *L. speciosa* leaf extracts- methanol, ethanol, acetone, and n-hexane in 10 mL of their respective solvents. Ascorbic acid was used as a positive control. The reaction mixtures were vortexed and incubated in the dark for 30 minutes. Various concentrations of the extracts (20, 40, 80, 160, 320, and 500 μg/mL) were used to monitor the transition of oxidized DPPH (initially violet) to reduced DPPH (final product, yellow). Absorbance readings were recorded at 517 nm. The percentage of scavenging activity was calculated using the following formula:


Scavenging Activity (%)=(Absorbance of control−Absorbance of sample/Absorbance of control)×100

The percentage of scavenging activities was plotted against the corresponding concentrations to determine the IC_50_ value using a nonlinear regression model with four parameters.

### ABTS free radical scavenging assay

The antioxidant activity of the *L. speciosa* leaf extracts was determined using the ABTS radical cation decolorization assay [36]. The ABTS^•+^ radical cation was prepared by combining 7 mM ABTS with 2.45 mM potassium persulfate in a glass tube. The mixture was left to stand at room temperature in the dark for 12–16 hours to allow the accumulation of the ABTS^•+^ radical cation. ABTS^•+^ exhibits a stable blue-green fluorescence, which diminishes upon reaction with hydrogen-donating antioxidants. For the assay, 1 mL of *L. speciosa* extracts at concentrations of 20, 40, 80, 100, and 150 μg/mL was mixed with 3 mL of ABTS^•+^ solution. The absorbance of the reaction mixture was measured at 734 nm to monitor the reduction in color intensity. Ascorbic acid was used as a positive control. A dose-response nonlinear regression curve was generated by plotting the percentage of scavenging activity against the extract concentrations to determine the IC_50_ value.

### Superoxide radical scavenging assay

The superoxide radical scavenging activity of *L. speciosa* was determined by the riboflavin-light-NBT (nitro blue tetrazolium) assay [37]. This assay measures the ability of superoxide dismutase (SOD) to neutralize superoxide radicals generated during the reaction of riboflavin with nitro blue tetrazolium (NBT) under light, leading to formazan formation [38]. The reaction mixture contained 50 mM phosphate buffer (pH 7.6), 20 μg riboflavin, 12 mM EDTA, and 0.1 mg NBT in a total volume of 3 mL. The reaction was initiated by exposing the mixture to a fluorescent lamp for 90 seconds, both with and without the addition of *L. speciosa* extracts at concentrations of 20, 40, 80, 160, and 300 μg/mL. The reduction in formazan formation was quantified by measuring the absorbance of the solution at 590 nm. Ascorbic acid was used as a positive control. The percentage of superoxide scavenging activity was calculated as follows:


$$\text{Scavenging}\;\text{activity}\;(\%)=({\text{A}}_{\text{0}}–{\text{A}}_{\text{1}}/{\text{A}}_{\text{0}})\times \text{100}$$

where A_0_ is the absorbance of the distilled water and A_1_ is an absorbance in the presence of the extract. A dose-response curve was generated using nonlinear regression, plotting scavenging activity percentages relative to extract concentrations. The IC_50_ value was derived from this curve.

### Nitric oxide free radical scavenging assay

The nitric oxide (NO) free radical of *L. speciosa* leaf extracts was determined by the modified Sreejayan et al. method [39]. Under anoxic conditions, sodium nitroprusside generates nitric oxide (NO), which reacts with oxygen to form nitrite ions. These ions are detected using the Griess reagent, where lower NO production indicates stronger scavenging activity by the extracts. 2 μL of sodium nitroprusside standard solution (5 mM) was aliquoted into tubes containing phosphate-buffered saline (PBS). *L. speciosa* leaf extracts (methanol, ethanol, acetone, and n-hexane) were added at concentrations of 20, 40, 80, 160, and 300 μg/mL. The mixture was incubated for 2 hours at room temperature (25°C). After incubation, the Griess reagent (1% sulfanilamide, 2% phosphoric acid, and 0.1% naphthyl ethylenediamine dihydrochloride) was added to the reaction mixture. This facilitated the formation of a chromophore through nitrite-sulfanilamide diazotization. The optical density of each sample was measured at 546 nm. Ascorbic acid was used as a positive control. The nitric oxide scavenging activity was calculated using the following formula:


$$\text{Scavenging}\;\text{activity}\;(\%)=({\text{A}}_{\text{0}}–{\text{A}}_{\text{1}}/{\text{A}}_{\text{0}})\times \text{100}$$

where A_0_ is the absorbance of the control and A_1_ is the absorbance of the sample extract. A nonlinear regression dose-response curve was plotted and IC_50_ value was determined from this curve.

### Brine shrimp lethality assay

*L. speciosa* leaf extract cytotoxicity was determined using the brine shrimp lethality assay [40]. Brine shrimp eggs were hatched in artificial seawater (3.8% NaCl solution) in a divided hatching aquarium tank (pH 7). A light source was positioned near the eggs to attract the hatched nauplii. After 48 hours of incubation, brine shrimp nauplii (*Artemia salina*) were collected for the experiment. To ensure the reliability of the test results, both experimental and control groups were established. 2 mg of *L. speciosa* methanol leaf extract was dissolved in 100 µL of dimethyl sulfoxide (DMSO) to prepare a stock solution of 20 mg/mL. The extract was diluted in artificial seawater to achieve concentrations of 25, 50, 100, 200, 400, and 800 μg/mL. Twenty nauplii were placed in each experimental bottle containing the prepared concentrations. The nauplii were incubated at room temperature for 24 hours. After incubation, the surviving nauplii were counted using a hand lens, and the percentage of mortality at each concentration was calculated. The LC_50_ value was determined by plotting the post-treatment concentration against mortality and performing a probit nonlinear regression analysis.

### Antibacterial activity

An antimicrobial activity of *L. speciosa* methanol extract was carried out using the agar disk diffusion method [41]. A stock solution was prepared by dissolving 0.1 g of the methanol extract in 100 mL of methanol, resulting in a final concentration of 100 mg/mL. This stock solution was further diluted to 1 mg/mL to serve as the working solution. Specimens were pre-moistened by applying 20 µL of each dilution, with 5 µL carefully applied to both sides of the disks. The disks were allowed to dry between applications to ensure homogeneous impregnation. Methanol and water-soaked disks were used as negative controls. Bacterial strains were used in the tests: *Escherichia coli* (ATCC 25922), *Salmonella enterica* (ATCC 14028), *Bacillus subtilis* (ATCC 6633), *Listeria monocytogenes* (ATCC 19115), and *Staphylococcus aureus* (ATCC 25923). These strains were obtained from the Institute of Nutrition and Food Science, University of Dhaka, Bangladesh. The strains were cultured on nutrient agar and stored at 4°C. Before the experiment, nutrient agar plates were inoculated with a bacterial suspension at a concentration of approximately 1 × 10^8^ CFU/mL. Small (6 mm in diameter) wells were made aseptically in the agar using a sterile cork borer, and disks were placed on the bacterial lawn. The plates were incubated at 37°C for 24 hours, and the antibacterial activity was determined from the inhibition zone (IZ) around the disks. To further characterize the antibacterial activity, the minimum inhibitory concentration (MIC) and minimum bactericidal concentration (MBC) were determined. Antibacterial activity was measured in millimeters (mm), with ampicillin disks serving as positive controls for all tested bacterial strains.

### Antifungal activity

The antifungal effectiveness of *L. speciosa* methanol leaf extract was tested using a well-diffusion technique [42]. The fungal strains selected for the study included *Aspergillus niger* (ATCC 16404), *Saccharomyces cerevisiae* (ATCC 9763), *Candida albicans* (ATCC 10231), and *Cladosporium sp.* (ATCC 16022). These strains were preserved on appropriate agar media to support their growth. Sabouraud dextrose agar (SDA) plates were prepared and streaked with a fungal suspension at a concentration of approximately 1 × 10^6^ colony-forming units per milliliter (CFU/mL). Wells (6 mm in diameter) were made using a sterile cork borer in the agar. A stock solution of the methanol extract of *L. speciosa* was prepared at 100 mg/mL and diluted to a working concentration of 1 mg/mL. Each well was loaded with the extract solution, and the plates were incubated at 28°C for 48 hours. After incubation, the antifungal activity was assessed by measuring the diameter of the inhibition zones around the wells using a digital caliper. We also quantitatively assessed antifungal activity by determining both the minimum inhibitory concentration (MIC) and minimum fungicidal concentration (MFC) values. Nystatin disks were included in each plate as a positive control.

### α-amylase inhibitory assay

Determination of α-amylase inhibitory activity of *L. speciosa* leaf extracts was carried out using the DNSA modified method [43]. Stock solutions with concentrations of 50, 100, 200, 400, and 500 µg/mL were prepared from the extracts using 10% DMSO. These solutions were mixed with a buffer solution consisting of Na_2_HPO_4_/NaH_2_PO_4_ (0.02 M) and NaCl (0.006 M) at pH 6.9. For the assay, 200 μL of α-amylase solution (2 units/mL) was combined with 200 μL of the extract and incubated at 30°C for 10 minutes. Subsequently, 200 μL of 1% starch solution was added, and the mixture was incubated for an additional 3 minutes. The reaction was terminated by adding 200 μL of DNSA reagent, followed by heating the solution to 85–90°C for 10 minutes. After cooling, the solution was diluted to 5 mL with distilled water, and the absorbance was measured at 540 nm. Acarbose was used as a positive control. The α-amylase inhibition (%) was calculated as:


$$\text{Inhibitions}\;(\%)=({\text{A}}_{\text{0}}–{\text{A}}_{\text{1}}/{\text{A}}_{\text{0}})\times \text{100}$$

where A_0_ is the absorbance of the control and A_1_ is the absorbance of the sample extracts. The IC_50_ value was determined by plotting inhibition percentages against concentrations using nonlinear regression analysis with four variables.

### α-glucosidase inhibition assay

The inhibitory effect of *L. speciosa* leaf extracts on α-glucosidase was determined the following protocol of Kazeem et al. [44]. A substrate solution of p-NPG (p-nitrophenyl glucopyranoside) was prepared in 20 mM phosphate buffer (pH 6.9). For the assay, 100 μL of the extract was preincubated with 50 μL of the extract at varying concentrations for 10 minutes. Subsequently, 50 μL of p-NPG substrate was added to initiate the reaction. Next, 20 μL of phosphate buffer (pH 6.9) was introduced, and the mixture was incubated at 37°C for 20 minutes. The reaction was terminated by adding 2 mL of 0.1 M Na_2_CO_3_, which facilitated the release of yellow p-nitrophenol. The absorbance of the released p-nitrophenol was quantified spectrophotometrically at 405 nm. The α-glucosidase inhibitory activity was calculated as follows:


$$\text{Inhibitions}\;(\%)=({\text{A}}_{\text{0}}–{\text{A}}_{\text{1}}/{\text{A}}_{\text{0}})\times \text{100}$$

where A_0_ is the absorbance of the control and A_1_ is the absorbance of the sample extracts. The IC_50_ value was determined by plotting inhibition percentages against concentrations.

### *In silico* pharmacological activity prediction

The biological activity profiles of major phytochemicals identified in *L. speciosa* were predicted using the Prediction of Activity Spectra for Substances (PASS) online platform (http://www.way2drug.com/PassOnline/). The analysis encompassed six principal compounds: corosolic acid, ellagic acid, quercetin, gallic acid, γ-sitosterol, and lagerstroemin. The PASS algorithm was employed to predict probable activity (Pa) and probable inactivity (Pi) for multiple therapeutic targets of interest, including anticancer, anti-inflammatory, antiseptic, free radical scavenging, lipid peroxidase inhibition, insulin promotion, and antioxidant properties.

### *In silico* prediction of pharmacokinetic parameters using SwissADME

ADME (Absorption, Distribution, Metabolism and Excretion) and drug-like characteristics of eight major bioactive compounds identified in *L. speciosa* were predicted using the SwissADME web-based platform (http://www.swissadme.ch/). The selected compounds- corosolic acid, ellagic acid, quercetin, γ-sitosterol, gallic acid, ursolic acid, phytol, and campesterol were chosen based on their documented abundance in *L. speciosa* leaf extracts from previous phytochemical studies. Drug-likeness assessment was performed according to Lipinski’s rule of five criteria, which evaluate molecular weight, lipophilicity, hydrogen bond donors, hydrogen bond acceptors and topological polar surface area (TPSA). This analysis provided valuable predictions concerning oral bioavailability and therapeutic viability of the target phytochemicals.

### *In silico* prediction of toxicological properties using AdmetSAR

The toxicological properties of major bioactive compounds identified in *L. speciosa* leaf extracts were evaluated using the AdmetSAR (Absorption, Distribution, Metabolism, Excretion, and Toxicity Structure-Activity Relationship) online prediction platform (https://lmmd.ecust.edu.cn/admetsar1/predict/). Six predominant compounds were selected for analysis: corosolic acid, ellagic acid, quercetin, γ-sitosterol, gallic acid, and phytol based on their reported abundance in *L. speciosa* extracts. Comprehensive toxicity profiling states multiple parameters, including AMES toxicity, carcinogenicity potential, honeybee toxicity, acute oral toxicity, fish toxicity, rat acute toxicity, and biodegradation properties.

### *In silico* molecular docking analysis

#### Preparation of ligands.

Major bioactive compounds commonly found in *L. speciosa* leaf extracts were selected for ligand preparation. The SMILES (Simplified Molecular Input Line Entry System) strings of Corosolic acid, Ellagic acid, Gallic acid, Quercetin, Lagerstroemin, and γ-Sitosterol were retrieved from the PubChem database and used as input for web-based molecular modeling using SwissDock.

#### Preparation of receptors/proteins.

The three-dimensional crystallographic structures of selected target proteins were downloaded from the RCSB Protein Data Bank (PDB) in PDB format [45]. The chosen targets included α-glucosidase (PDB ID: 3WY1), DNA gyrase B (PDB ID: 6KZV), β-lactamase (PDB ID: 1XPB), peroxiredoxin 6 (PDB ID: 5B6M), and peroxisome proliferator-activated receptor gamma (PPAR-γ; PDB ID: 2P4Y). These proteins were selected based on their relevance to antidiabetic, antimicrobial, and antioxidant pharmacological activities. The prepared receptor structures were docked with selected phytocompounds using SwissDock to predict ligand-protein interactions and assess pharmacological potential.

#### Molecular docking with attracting cavities using SwissDock.

*In silico* molecular docking was carried out to predict ligand-receptor interactions and identify pharmacologically relevant binding cavities. The docking studies were performed using the SwissDock web server (http://www.swissdock.ch) with the “attracting cavities” docking mode to enable high-precision predictions. Both receptor and ligand structures were submitted to SwissDock for analysis. The resulting docked complexes were further visualized using PyMOL for structural analysis. Key parameters, including the Attracting Cavities (AC) score, binding free energy (SwissParam score), and root-mean-square deviation (RMSD), were analyzed to assess docking accuracy and complex stability.

### Ethical clearance

The entire experimental design was carried out under the comprehensive ethical supervision of the Experimental Animal Committee at the Institute of Biological Sciences, University of Rajshahi. The study design was formally approved by the committee under approval number 225/322-IAMEBBC/IBSc.

### Statistical analysis

Data are presented as mean ± standard deviation (SD) for statistical assessments in triplicate. IC_50_ and LC_50_ values were calculated from the GraphPad Prism 10.4.1 version (Boston, MA, USA). Molecular docking results were visualized and analyzed using PyMOL version 3.1.1 (Schrodinger, LLC). Ordinary one-way and two-way analysis of variance (ANOVA) followed by Tukey’s multiple comparison tests were performed with significance set at *p < 0.05.

## Results

### Total phenolic and flavonoid content of *L. speciosa* leaf extracts

**Fig 1:**
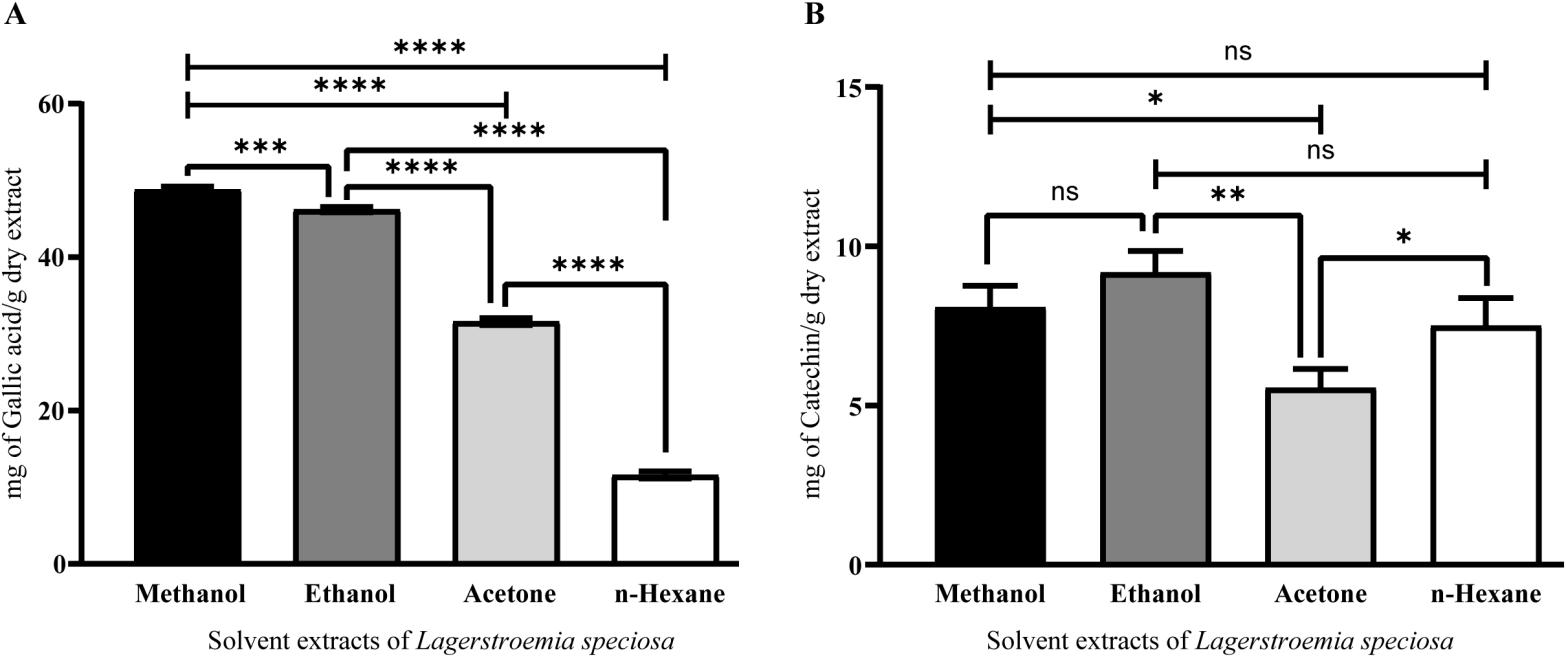
Quantification of total phenolic content (TPC) and total flavonoid content (TFC) in *L. speciosa* leaf extracts using different solvents. (A) TPC was determined using the Folin-Ciocalteu assay and expressed as milligrams of gallic acid equivalent (GAE) per gram of dry extract. Methanol and ethanol extracts exhibited significantly higher TPC values compared to acetone and n-hexane. (B) TFC was quantified using a standard colorimetric method and expressed as milligrams of catechin equivalent (CE) per gram of dry extract. Ethanol and methanol extracts showed the highest TFC, followed by n-hexane and acetone. Data are presented as mean ± SD from three independent experiments. Statistical significance was determined using one-way ANOVA followed by Tukey’s post-hoc test. Significant differences between solvents are indicated by asterisks (*p < 0.05, **p < 0.01, ***p < 0.001, ****p < 0.0001) and “ns” denoting not significant.

*L. speciosa* leaf extracts revealed a significant amount of phenolic and flavonoid phytochemicals. Methanol extracts exhibited the highest TPC at 48.7 ± 0.3 mg GAE/g, followed closely by ethanol extracts at 46.2 ± 0.2 mg GAE/g. Acetone and n-hexane extracts yielded significantly lower TPC values of 31.5 ± 0.4 mg GAE/g and 11.8 ± 0.3 mg GAE/g, respectively, highlighting the superior efficacy of polar solvents for phenolic extraction (**Fig 1A**). Ethanol extracts showed the highest TFC at 9.2 ± 0.6 mg CE/g, followed by methanol at 8.1 ± 0.7 mg CE/g, n-hexane at 7.5 ± 0.8 mg CE/g, and acetone at 5.6 ± 0.5 mg CE/g. We observed the lowest extraction efficiency in the acetone extract of *L. speciosa* leaves (**Fig 1B**).

### Total flavonol and proanthocyanidin content of *L. speciosa* leaf extracts

**Fig 2:**
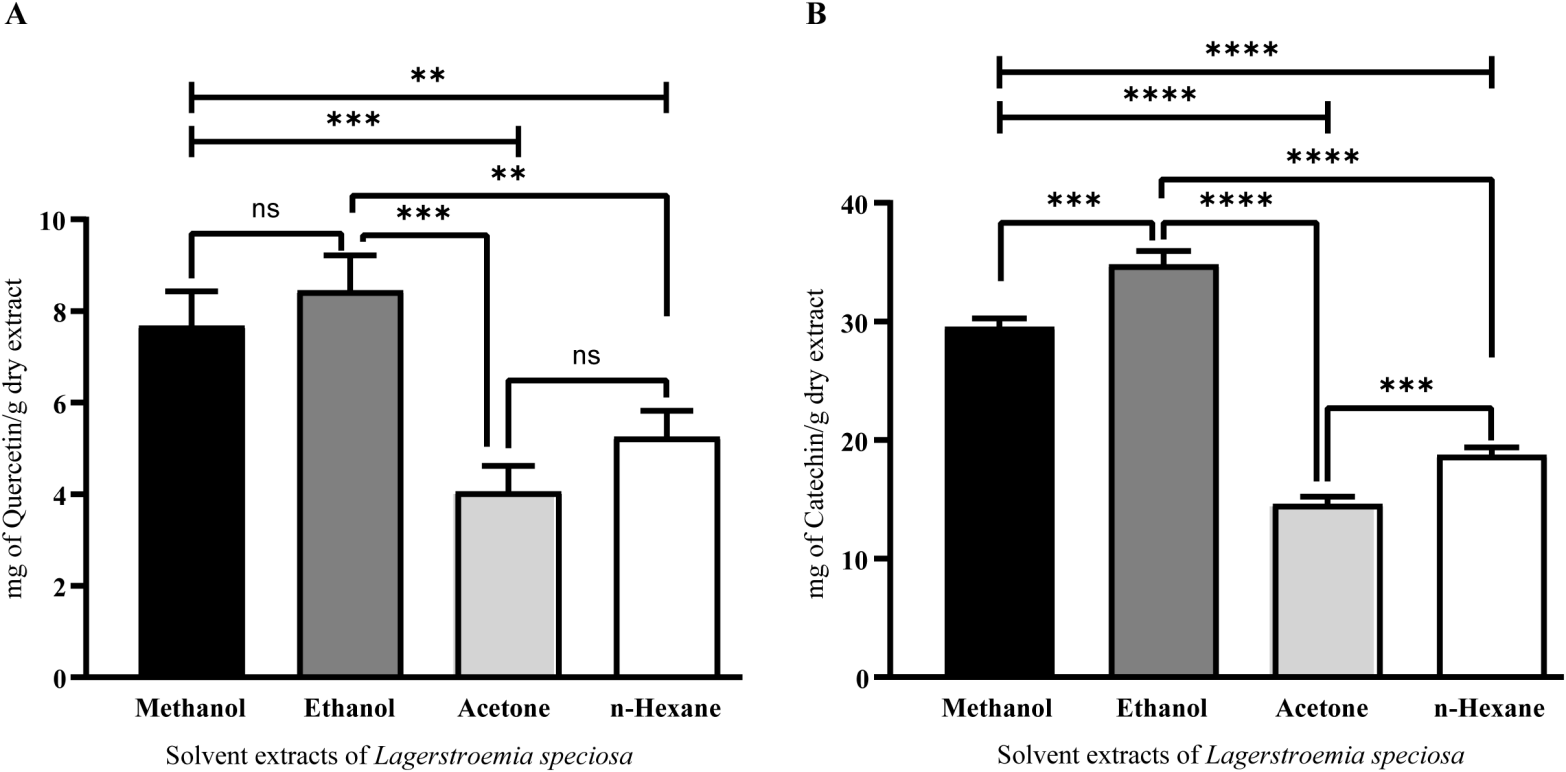
Quantification of total flavonol and proanthocyanidin content in *L. speciosa* leaf extracts using different solvents. (A) Total flavonol content was determined using aluminum chloride colorimetric method and expressed as milligrams of quercetin equivalent (QE) per gram of dry extract. Methanol and ethanol extracts exhibited significantly higher flavonol content than acetone and n-hexane. (B) Total proanthocyanidin content was quantified using the vanillin-HCl assay and expressed as milligrams of catechin equivalent (CE) per gram of dry extract. Ethanol extracts showed the highest proanthocyanidin content, followed by methanol, n-hexane, and acetone. Data are denoted as mean ± standard error (n = 3). Statistical significance was determined using one-way ANOVA followed by Tukey’s post-hoc test. Significant differences between solvents are indicated by asterisks (**p < 0.01, ***p < 0.001, ****p < 0.0001) and “ns” denoting not significant.

The leaf extracts of *L. speciosa* contained substantial amounts of flavonols and proanthocyanidins. Ethanol extracts exhibited the highest flavonol content (8.4 ± 0.7 mg QE/g), followed by methanol (7.8 ± 0.6 mg QE/g), n-hexane (5.2 ± 0.5 mg QE/g), and acetone extracts demonstrated the lowest content (4.1 ± 0.4 mg QE/g).These results emphasize the solvent-dependent extraction efficiency for flavonol (**Fig 2A**). Quantitative analysis revealed substantial levels of total proanthocyanidins in *L. speciosa* leaf extracts. Among the tested solvents, ethanol demonstrated the highest proanthocyanidin content (34.8 ± 1.2 mg CE/g), indicating its superior ability to extract these bioactive compounds. Methanol extracts also showed a relatively high proanthocyanidin concentration (29.4 ± 0.8 mg CE/g), suggesting that both ethanol and methanol are effective solvents for polyphenolic extraction. In contrast, n-hexane (19.2 ± 0.5 mg CE/g) and acetone (14.8 ± 0.4 mg CE/g) exhibited lower proanthocyanidin levels, likely due to differences in solvent polarity and their ability to solubilize these compounds. These findings highlight the role of solvent selection in optimizing the extraction of proanthocyanidins from *L. speciosa* leaves, with ethanol emerging as the most efficient solvent in this study (**Fig 2B**).

### DPPH and ABST free radical scavenging assay of *L. speciosa* leaf extracts

**Fig 3:**
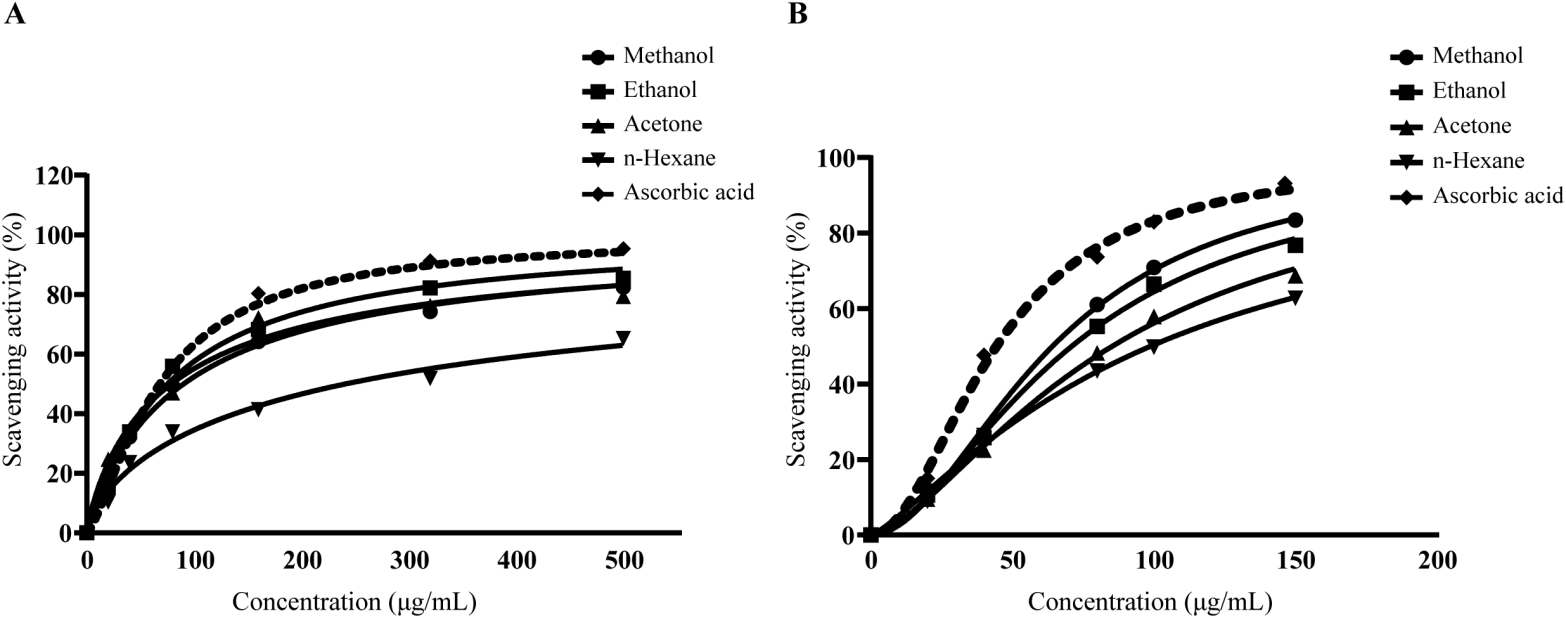
*L. speciosa* leaf extracts and their DPPH and ABTS free radical scavenging activity. **(A)** DPPH radical scavenging activity was evaluated at concentrations ranging from 0 to 500 µg/mL for *L. speciosa* leaf extracts and compared to ascorbic acid (positive control). Scavenging activity increased in a concentration-dependent manner. Ascorbic acid showed the highest inhibition, followed by ethanol, methanol, acetone, and n-hexane. **(B)** ABTS radical scavenging activity was assessed using the ABTS decolorization assay at concentrations ranging from 0 to 150 µg/mL. Ascorbic acid demonstrated the highest scavenging potential, followed by methanol, ethanol, acetone, and n-hexane. Data were analyzed using an inhibitor vs. dose-response four-variable parameter model to determine IC_50_ values. Results represent the mean ± standard deviation from three independent experiments (n = 3).

The DPPH radical scavenging activity was evaluated using *L. speciosa* extracts at concentrations ranging from 0 to 500 µg/mL. The ethanol extract demonstrated the highest scavenging activity in the DPPH assay, with inhibition ranging from 15.31 ± 0.64% at 20 µg/mL to 85.4 ± 0.46% at 500 µg/mL compared with methanol, acetone, and n-hexane extracts. The IC_50_ values for the extracts were determined to be 90.39 µg/mL for methanol, 75.53 µg/mL for ethanol, 78.92 µg/mL for acetone, and 241.59 µg/mL for n-hexane. The standard antioxidant, ascorbic acid, demonstrated the highest potency (68.16 µg/mL) among all extracts of *L. speciosa* (**Fig 3A**). In the ABTS assay, the antioxidant potential was assessed using *L. speciosa* extracts at concentrations ranging from 0 to 150 µg/mL. The methanol extract exhibited significant concentration-dependent scavenging activity, reaching 83.45 ± 0.22% inhibition at 150 µg/mL, while the n-hexane extract showed 62.75 ± 0.21% inhibition at the same concentration. Ascorbic acid, the positive control, achieved the highest scavenging activity at 93.13 ± 0.68% (**Fig 3B**). The methanol extract of *L. speciosa* showed an IC_50_ value of 64.19 µg/mL, followed by the ethanol (70.99 µg/mL), acetone (85.26 µg/mL), and n-hexane (98.78 µg/mL) extracts. Ascorbic acid had a stronger activity with an IC_50_ of 44.41 µg/mL. These results indicate that the methanol and ethanol extracts possess substantial free radical scavenging activity, although slightly lower potency than ascorbic acid.

### Superoxide and nitric oxide free radical scavenging assay of *L. speciosa* leaf extracts

**Fig 4:**
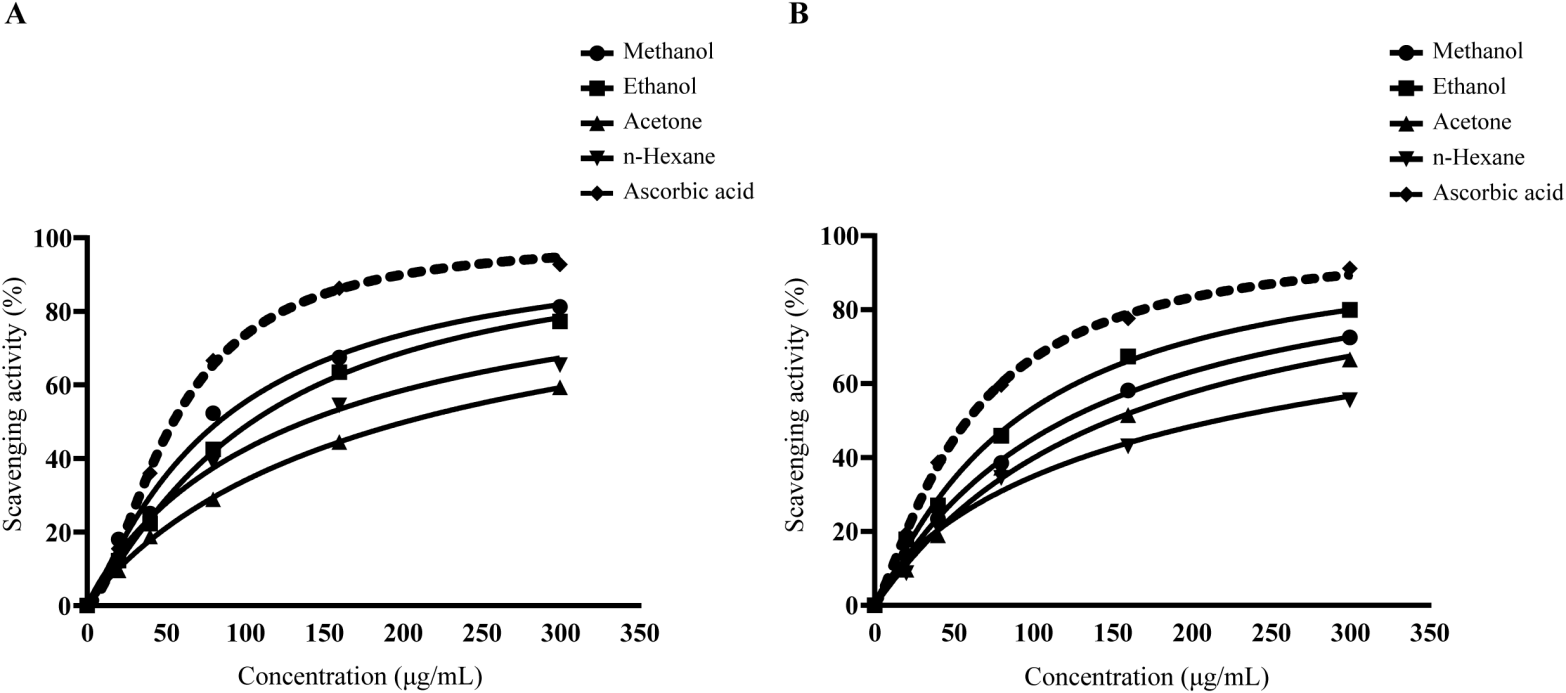
Superoxide and nitric oxide free radical scavenging activity of *L. speciosa* leaf extracts. (A) Superoxide radical scavenging activity of *L. speciosa* leaf extracts and ascorbic acid was measured using the riboflavin-light-nitro blue tetrazolium (NBT) reduction method at concentrations ranging from 0 to 300 µg/mL. Methanol extract exhibited the highest activity among the extracts (B) Nitric oxide (NO) radical scavenging activity of the extracts and ascorbic acid was assessed using the Griess reagent method at concentrations ranging from 0 to 300 µg/mL. Ethanol extract showed the highest activity among the extracts. Ascorbic acid demonstrated the highest scavenging activity compared to all solvent extracts. Data were analyzed using an inhibitor vs. dose-response four-variable parameter model to determine IC_50_ values. Results are expressed as mean ± SD of triplicate measurements from three biologically independent experiments.

In the superoxide assay, the scavenging potential was assessed at concentrations ranging from 0 to 300 µg/mL. The methanol extract demonstrated the highest scavenging activity, ranging from 18.02 ± 0.51% at 20 µg/mL to 81.21 ± 0.62% at 300 µg/mL. The ethanol extract followed, while the acetone and n-hexane extracts exhibited moderate to low activity. The standard antioxidant, ascorbic acid, achieved 92.73 ± 0.35% inhibition at 300 µg/mL. The half-maximal inhibitory concentration (IC_50_) values for superoxide anion radical scavenging demonstrated varying efficacies among the extracts. The methanol extract exhibited an IC_50_ value of 83.58 µg/mL, while the ethanol extract required 104.85 µg/mL to achieve comparable inhibition. The acetone and n-hexane extracts displayed relatively lower antioxidant potencies with IC_50_ values of 202.17 µg/mL and 138.65 µg/mL, respectively. The reference antioxidant, ascorbic acid, demonstrated superior scavenging capacity with an IC_50_ of 54.84 µg/mL (**Fig 4A**). In the nitric oxide assay, the ethanol extract showed the highest inhibition at 79.94 ± 0.34% at 300 µg/mL, followed by the methanol extract at 72.45 ± 0.14%. The nitric oxide radical scavenging potential of the extracts was expressed as IC_50_ values, demonstrated varying degrees of efficacy across different extraction solvents. The ethanol extract exhibited superior scavenging activity (89.09 μg/mL), followed by methanol (120.45 μg/mL), acetone (149.18 μg/mL), and n-hexane extracts (216.33 μg/mL). While all extracts demonstrated concentration-dependent inhibition, the reference standard ascorbic acid displayed significantly higher potency (58.58 μg/mL). These findings indicate that medium-polarity solvents more effectively extracted compounds with nitric oxide scavenging capabilities (**Fig 4B**).

### Brine shrimp lethality assay of *L. speciosa* leaf extracts

**Fig 5:**
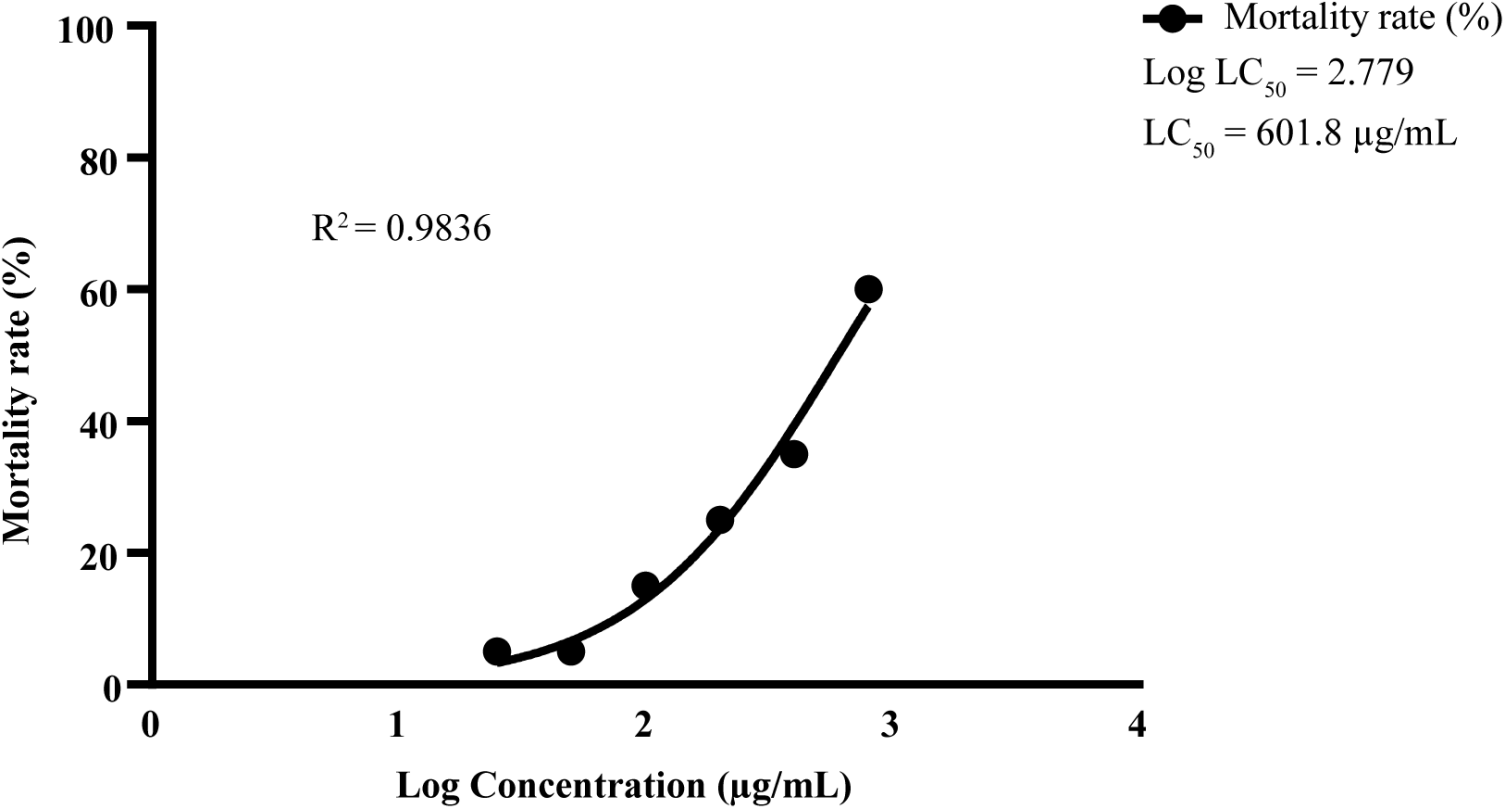
Brine shrimp lethality assay of *L. speciosa* leaf extracts. Cytotoxicity of *L. speciosa* methanol leaf extract was evaluated using the brine shrimp lethality assay (BSLA). Extracts, prepared in dimethyl sulfoxide (DMSO), were tested at 0 - 800 µg/mL. The concentration-response relationship was analyzed using nonlinear regression (coefficient of determination, R^2^ = 0.9836), yielding an LC_50_ value of 601.8 μg/mL. According to Meyer’s toxicity index, the extract demonstrates moderate cytotoxic potential (LC_50_ between 500 - 1000 μg/mL). Data represent the mean ± standard deviation from three independent experiments (n = 3).

In the brine shrimp lethality assay (BSLA), concentrations ranging from 0 to 800 µg/mL were employed to assess the cytotoxic effects of *L. speciosa*. The assay revealed a concentration-dependent increase in the mortality of *Artemia salina* nauplii, with mortality rates rising from 5% at 25 µg/mL to 60% at 800 µg/mL. Nonlinear regression analysis demonstrated a strong correlation between extract concentration and mortality, as indicated by a high coefficient of determination (R^2^ = 0.9836). The LC_50_ value, representing the concentration required to induce 50% mortality in the nauplii population, was determined to be about 601.8 µg/mL. The high R^2^ value highlights a clear dose-response relationship, suggesting that the cytotoxic activity is likely attributable to bioactive compounds present in the extract. These findings indicate that the methanol extract of *L. speciosa* exhibits moderate cytotoxic effects (**Fig 5**).

### Antibacterial activity of *L. speciosa* leaf extracts

The antibacterial activity of methanol-derived *L. speciosa* leaf extract was evaluated against various bacterial species, as summarized in **Table 1**. The methanol extract (1 mg/disk) demonstrated inhibitory effects, with mean inhibition zones of 17 ± 0.34 mm for *Escherichia coli*, 14 ± 0.97 mm for *Salmonella enterica*, 15 ± 0.40 mm for *Bacillus subtilis*, 16 ± 0.80 mm for *Listeria monocytogenes*, and 15 ± 0.10 mm for *Staphylococcus aureus*. In comparison, the standard antibiotic ampicillin (20 µg/disk) exhibited significantly larger inhibition zones: 25 ± 1.50 mm for *Escherichia coli*, 26 ± 0.46 mm for *Salmonella enterica*, 27 ± 0.40 mm for *Bacillus subtilis*, 29 ± 0.83 mm for *Listeria monocytogenes*, and 28 ± 1.20 mm for *Staphylococcus aureus*. MIC values ranged from 250 to 500 µg/mL, while MBC values ranged from 500 to 1000 µg/mL. Specifically, the MBC/MIC ratios were as follows: *E. coli* (MIC: 250 µg/mL, MBC: 500 µg/mL, ratio = 2), *S. enterica* (MIC: 500 µg/mL, MBC: 1000 µg/mL, ratio = 2), *B. subtilis* (MIC: 250 µg/mL, MBC: 500 µg/mL, ratio = 2), *L. monocytogenes* (MIC: 250 µg/mL, MBC: 500 µg/mL, ratio = 2), and *S. aureus* (MIC: 250 µg/mL, MBC: 500 µg/mL, ratio = 2). All MBC/MIC ratios were ≤ 2, indicating that the methanol extract exhibited bactericidal activity against the tested strains. Methanol extract of *L. speciosa* displayed substantial antibacterial activity. Their efficacy was considerably lower than standard antibiotics (ampicillin) against all tested bacterial strains. The highest antibacterial activity of the extract was observed against *Escherichia coli*, while the lowest activity was recorded against *Salmonella enterica*. All antimicrobial activity differences between the methanol extract and ampicillin control were statistically significant (paired t-test, p < 0.0001).

**Table 1:** Antibacterial activity of the methanol extract of *L. speciosa* leaves and ampicillin (control) disk.

Name of the bacteria (Code)	Diameter of zone of inhibition mean (mm) ± SD				
	*L. speciosa* extract (1 mg/disk)	Ampicillin (20 µg/disk)	MIC (µg/mL)	MBC (µg/mL)	MBC/MIC
*Escherichia coli* (ATCC 25922)	17 ± 0.34	25 ± 1.50	250	500	2
*Salmonella enterica* (ATCC 14028)	14 ± 0.97	26 ± 0.46	500	1000	2
*Bacillus subtilis* (ATCC 6633)	15 ± 0.40	27 ± 0.40	250	500	2
*Listeria monocytogenes* (ATCC 19115)	16 ± 0.80	29 ± 0.83	250	500	2
*Staphylococcus aureus* (ATCC 25923)	15 ± 0.10	28 ± 1.20	250	500	2

*Note*. Values represent the mean diameter of the inhibition zone (mm) ± standard deviation (SD) from three independent experiments (n = 3). MIC: Minimum Inhibitory Concentration; MBC: Minimum Bactericidal Concentration. The MBC/MIC ratio was used to evaluate bactericidal activity, with a ratio ≤4 indicating bactericidal potential. All differences in inhibition zones between the extract and standard were statistically significant (paired t-test, *p* < 0.0001).

### Antifungal activity of *L. speciosa* leaf extracts

The antifungal activity of *L. speciosa* methanol leaf extracts was evaluated against several fungal species using the agar disk diffusion method, as summarized in **Table 2**. At a concentration of 1 mg/disk, the methanol extract exhibited inhibitory effects against all tested fungal species, with mean inhibition zones of 12 ± 0.11 mm for *Aspergillus niger*, 13 ± 0.88 mm for *Saccharomyces cerevisiae*, 12 ± 0.92 mm for *Candida albicans*, and 14 ± 0.35 mm for *Cladosporium sp*. In comparison, the standard antifungal agent nystatin (30 µg/disk) demonstrated significantly larger inhibition zones: 22 ± 0.32 mm for *Aspergillus niger*, 21 ± 0.19 mm for *Saccharomyces cerevisiae*, 23 ± 0.58 mm for *Candida albicans*, and 20 ± 0.94 mm for *Cladosporium sp*. The MIC values for the methanol extract ranged from 300 to 600 µg/mL, while MFC values ranged from 600 to 1200 µg/mL. Specifically, the MFC/MIC ratios were: *A. niger* (MIC: 600 µg/mL, MFC: 1200 µg/mL, ratio = 2), *S. cerevisiae* (MIC: 300 µg/mL, MFC: 600 µg/mL, ratio = 2), *C. albicans* (MIC: 400 µg/mL, MFC: 800 µg/mL, ratio = 2), and *Cladosporium* sp. (MIC: 300 µg/mL, MFC: 600 µg/mL, ratio = 2). All MFC/MIC ratios were ≤ 2, indicating fungicidal activity of the extract against the tested strains. The methanol extract of *L. speciosa* displayed significant antifungal activity. Their efficacy was markedly lower than standard antibiotics (nystatin) across all tested fungal strains. Statistical analysis using paired t-tests revealed that all extracts were significant compared to the reference standard (**p < 0.005).

**Table 2:** Antifungal activity of the methanol extract of *L. speciosa* leaves using the agar disk diffusion method.

Fungi Species (Code)	Diameter of zone of inhibition mean (mm) ± SD				
	*L. speciosa* extract (1 mg/disk)	Nystatin (30 µg/disk)	MIC (µg/mL)	MFC (µg/mL)	MFC/MIC
*Aspergillus niger* (ATCC 16404)	12 ± 0.11	22 ± 0.32	600	1200	2
*Saccharomyces cerevisiae* (ATCC 9763)	13 ± 0.88	21 ± 0.19	300	600	2
*Candida albicans* (ATCC 10231)	12 ± 0.92	23 ± 0.58	400	800	2
*Cladosporium sp.* (ATCC 16022)	14 ± 0.35	20 ± 0.94	300	600	2

*Note*. Values represent the mean diameter of the zone of inhibition (mm) ± standard deviation (SD) from three independent experiments (n = 3). MIC: Minimum Inhibitory Concentration; MFC: Minimum Fungicidal Concentration. MFC/MIC ratios ≤4 indicate fungicidal activity. A paired t-test was performed with nystatin (30 µg/disk) serving as the control. Statistical significance was considered at *p < 0.05, and two-tailed comparisons resulted in **p < 0.01.

### Enzyme inhibition assays of *L. speciosa* leaf extracts

**Fig 6:**
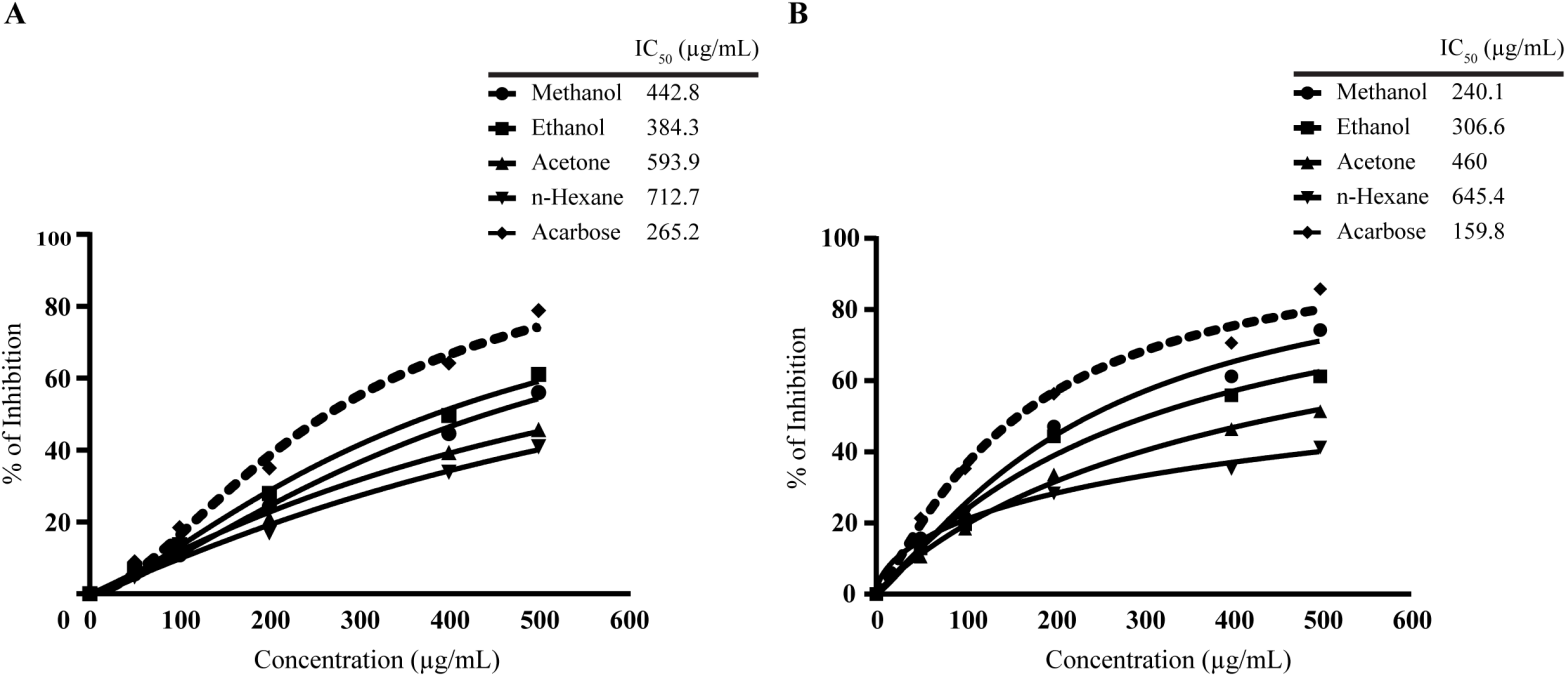
Enzyme inhibitory activities of *L. speciosa* leaf extracts prepared using different solvents. **(A)** α-amylase and **(B)** α-glucosidase inhibitory activities of extracts obtained with methanol, ethanol, acetone, and n-hexane. Acarbose served as positive control. Enzyme inhibition was evaluated across a concentration range of 0 - 500 μg/mL. Among the tested extracts, the ethanol extract exhibited the most potent α-amylase inhibition (IC_50_ = 240.1 μg/mL), while the methanol extract showed the highest α-glucosidase inhibitory activity (IC_50_ = 442.8 μg/mL). Dose response curves were fitted using a four-parameter variable slope model (inhibitor vs. response) to determine IC_50_ values. Statistical significance was robust across all experiments, with *p* < 0.0001.

The enzyme inhibitory properties of *L. speciosa* leaf extracts were assessed against α-amylase and α-glucosidase. The inhibitory effects of methanol, ethanol, acetone, and n-hexane extracts were evaluated at concentrations ranging from 0 to 500 µg/mL using nonlinear regression analysis. Acarbose was used as the reference standard. All extracts exhibited concentration-dependent inhibition in both enzyme assays. In the α-amylase assay, the ethanol achieved the highest inhibition (61.0 ± 0.6%) at 500 µg/mL, followed by methanol (56.0 ± 0.9%), acetone (45.7 ± 0.5%), and n-hexane (41.0 ± 0.3%). In comparison, acarbose demonstrated superior inhibition at 78.9 ± 0.5%. The IC_50_ values further supported these findings, with ethanol showing the lowest IC_50_ (384.3 µg/mL), followed by methanol (442.8 µg/mL), acetone (593.9 µg/mL), and n-hexane (712.7 µg/mL). Acarbose demonstrated the most potent activity with an IC_50_ of 265.2 µg/mL (**Fig 6A**). In the α-glucosidase assay, methanol extract yielded approximately 74.3 ± 1.0% inhibition at 500 µg/mL, increasing from 15.7 ± 0.9% at 50 µg/mL. Ethanol extract achieved 61.2 ± 1.4%, followed by acetone (51.4 ± 0.8%) and n-hexane (41.3 ± 1.2%) at 500 µg/mL. Acarbose demonstrated the highest inhibition, peaking at 85.8 ± 0.8% at 500 µg/mL, surpassing the extracts of *L. speciosa* leaves. The IC_50_ value of acarbose was estimated to be approximately 159.76 μg/mL. The IC_50_ value for the methanol extract was significantly lower (240.11 µg/mL) compared to ethanol (306.61 µg/mL), acetone (460.04 µg/mL), and n-hexane (645.39 µg/mL) (**Fig 6B**). These findings suggest that ethanol and methanol are the most suitable solvents for *L. speciosa* extraction due to their higher yield of bioactive compounds.

### *In silico* analysis of pharmacological potential

The PASS algorithm predicted probable activity (Pa) and probable inactivity (Pi) scores for a range of pharmacological targets, with Pa > 0.7 indicating robust activity. This computational analysis revealed diverse bioactivities among the evaluated compounds (**Table 3**). Among the compounds analyzed, corosolic acid emerged as a particularly promising candidate, demonstrating exceptional potential as an insulin promoter (Pa = 0.924). Enzymatic inhibitory properties were observed for ellagic acid, particularly as a ubiquinol-cytochrome-c reductase inhibitor (Pa = 0.907) and a peroxidase inhibitor (Pa = 0.861). Quercetin showed exceptional activity as a peroxidase inhibitor (Pa = 0.962) coupled with significant MAP kinase stimulation (Pa = 0.933). The analysis further revealed gallic acid’s dual functionality as a glutathione thiolesterase inhibitor (Pa = 0.944) and antiseptic agent (Pa = 0.910). γ-Sitosterol was predicted to be active in lipid regulation and chemoprevention, with Pa values up to 0.960. Moreover, the prediction revealed that lagerstroemin possesses potent histidine kinase inhibitory activity (Pa = 0.924) and antineoplastic potential (Pa = 0.893).

**Table 3:** Biological activities predicted for the major compounds of *L. speciosa* using the Prediction of Activity Spectra for Substances (PASS) online.

Compound Name	Biological Properties	Pa	Pi
Corosolic acid	Insulin promoter	0.924	0.002
	Transcription factor NF kappa B stimulant	0.922	0.001
	Anti-inflammatory	0.899	0.004
	Apoptosis agonist	0.895	0.004
	Nitric oxide antagonist	0.853	0.002
	Lipid peroxidase inhibitor	0.816	0.003
Ellagic acid	Ubiquinol-cytochrome-c reductase inhibitor	0.907	0.005
	Peroxidase inhibitor	0.861	0.004
	Kinase inhibitor	0.849	0.004
	Glutathione thiolesterase inhibitor	0.782	0.009
	Anti-inflammatory	0.749	0.010
	Apoptosis agonist	0.709	0.014
Quercetin	Peroxidase inhibitor	0.962	0.001
	MAP kinase stimulant	0.933	0.001
	NADPH oxidase inhibitor	0.928	0.002
	Apoptosis agonist	0.887	0.005
	Antioxidant	0.872	0.003
	Free radical scavenger	0.811	0.003
Gallic acid	Glutathione thiolesterase inhibitor	0.944	0.002
	Sugar-phosphatase inhibitor	0.941	0.003
	Glucan endo-1,6-beta-glucosidase inhibitor	0.933	0.002
	Antiseptic	0.910	0.003
	Superoxide dismutase inhibitor	0.898	0.004
	Peroxidase inhibitor	0.891	0.003
γ-Sitosterol	Antihypercholesterolemic	0.960	0.002
	Hypolipemic	0.924	0.004
	Oxidoreductase inhibitor	0.886	0.003
	Sulfotransferase substrate	0.832	0.004
	Chemopreventive	0.831	0.003
	Caspase 3 stimulant	0.806	0.005
Lagerstroemin	Histidine kinase inhibitor	0.924	0.002
	Antineoplastic	0.893	0.005
	Antioxidant	0.783	0.004
	Anti-inflammatory	0.786	0.008
	Free radical scavenger	0.767	0.003
	Beta glucuronidase inhibitor	0.765	0.003

*Note.* Pa = Probable activity, Pi = Probable inactivity, Pa > 0.7

### *In silico* assessment of pharmacokinetic properties

The pharmacokinetic profiles and drug-likeness parameters for key bioactive compounds from *L. speciosa* are detailed in **Table 4**. Drug-likeness was assessed based on Lipinski’s Rule of Five which predicts favorable oral bioavailability. Ellagic acid, quercetin, and gallic acid fully complied with Lipinski’s rule of five, demonstrating favorable molecular weight, lipophilicity (LogP), hydrogen bond donor and acceptor counts, and topological polar surface area. These compounds also showed high gastrointestinal (GI) absorption potential and comparable bioavailability scores ranging from 0.55 to 0.56. In contrast, corosolic acid, γ-sitosterol, ursolic acid, phytol, and campesterol each violated one Lipinski’s rule of five primarily due to lipophilicity (LogP). Despite this, corosolic acid retained high GI absorption and a moderate bioavailability score of 0.56. Conversely, γ-sitosterol, ursolic acid, phytol, and campesterol were predicted to have low GI absorption and varied bioavailability scores. The number of rotatable bonds among compounds ranged from 0 (ellagic acid) to 13 (phytol), reflecting varying degrees of molecular flexibility.

**Table 4:** ADME and Drug-likeness evaluation of selected compounds from *L. speciosa.*

Compound	MW (g/mol)	LogP	TPSA (Å^2^)	HBD	HBA	nRB	Lipinski Violations	GI Absorption	Bioavailability Score
Corosolic acid	472.7	5.06	77.76	3	4	1	1 (LogP)	High	0.56
Ellagic acid	302.2	1.0	141.34	4	8	0	0	High	0.55
Quercetin	302.24	1.23	131.36	5	7	1	0	High	0.55
γ-Sitosterol	414.71	7.24	20.23	1	1	6	1 (LogP)	Low	0.55
Gallic acid	170.12	0.21	97.99	4	5	1	0	High	0.56
Ursolic acid	456.7	5.93	57.53	2	3	1	1 (LogP)	Low	0.85
Phytol	296.53	6.25	20.23	1	1	13	1 (LogP)	Low	0.55
Campesterol	400.68	6.92	20.23	1	1	5	1 (LogP)	Low	0.55

*Note*. MW: Molecular weight, Log P: Lipophilicity, TPSA: Topological polar surface area, HBD: Hydrogen bond donor, HBA: Hydrogen bond acceptor, nRB: Number of rotatable bond and GI: Gastrointestinal absorption.

### *In silico* assessment of toxicological properties

A comprehensive toxicological assessment was conducted on six major bioactive compounds extracted from *L. speciosa* leaves: corosolic acid, ellagic acid, quercetin, γ-sitosterol, gallic acid, and phytol. The predicted toxicological parameters are summarized in **Table 5**. All compounds demonstrated favorable safety profiles, with predictions indicating non-mutagenic properties (Non AMES toxic) and no carcinogenic potential. The evaluated compounds exhibited high toxicity to honeybees (LD_50_ < 100 µg/bee), indicating potential ecological risks for pollinator species. Gallic acid and phytol were predicted as readily biodegradable, while the other compounds were classified as not readily biodegradable. Regarding acute oral toxicity, ellagic acid and quercetin were categorized as Category II (50 mg/kg < LD_50_ < 500 mg/kg), whereas corosolic acid, gallic acid, and phytol were assigned to Category III (500 mg/kg < LD_50_ < 5000 mg/kg). γ-Sitosterol was classified under Category I (LD_50_ ≤ 50 mg/kg). The phytochemicals uniformly induced fathead minnow toxicity, with LC_50_ predictions consistently below 0.5 mmol/L. Predicted acute toxicity in rats ranged from 1.6146 for phytol to 3.0200 for quercetin.

**Table 5:** Toxicological properties of the identified compounds from L. speciosa.

Parameters	Compounds					
	Corosolic acid	Ellagic acid	Quercetin	γ-Sitosterol	Gallic acid	Phytol
AMES Toxicity	NAT	NAT	NAT	NAT	NAT	NAT
Carcinogens	NC	NC	NC	NC	NC	NC
Honeybee Toxicity	High HBT	High HBT	High HBT	High HBT	High HBT	High HBT
Biodegradation	Not ready biodegradable	Not ready biodegradable	Not ready biodegradable	Not ready biodegradable	Ready biodegradable	Ready biodegradable
Acute Oral Toxicity	III	II	II	I	III	III
Fish Toxicity	High FHMT	High FHMT	High FHMT	High FHMT	High FHMT	High FHMT
Rat Acute Toxicity	2.1021	2.6213	3.0200	2.6561	1.8670	1.6146

*Note*. NAT: Non-AMES Toxic; NC: Non-carcinogenic; HBT: Honey Bee Toxicity (compounds with LD_50_ < 100 μg/bee are classified as high acute HBT); FHMT: Fathead Minnow Toxicity (compounds with LC_50_ < 0.5 mmol/L are classified as high acute FHMT). Oral Toxicity categories: Category-I (LD_50_ ≤ 50 mg/kg); Category-II (50 mg/kg < LD_50_ < 500 mg/kg); Category-III (500 mg/kg < LD_50_ < 5000 mg/kg).

### *In silico* analysis of molecular docking interactions

**Fig 7:**
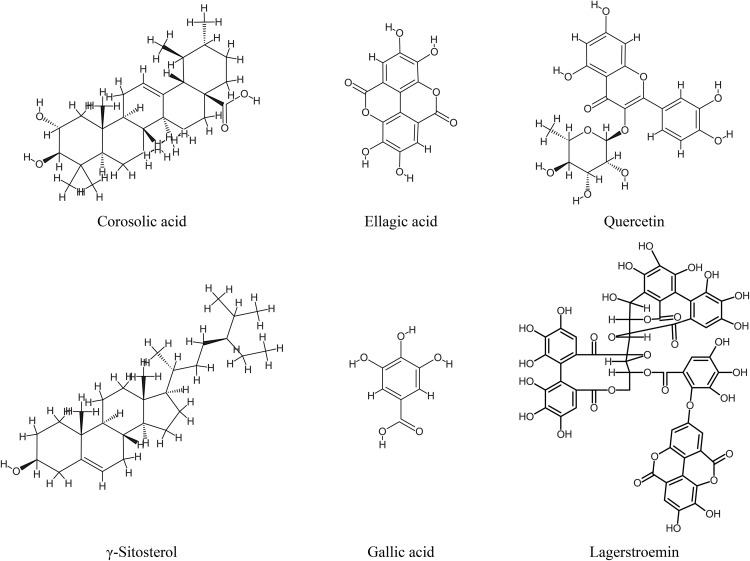
Chemical structures of the major compounds identified in *L. speciosa* leaves. The compounds depicted are Corosolic acid, Ellagic acid, Quercetin, γ-Sitosterol, Gallic acid, and Lagerstroemin. These compounds were selected based on their abundance and potential pharmacological relevance.

**Fig 8:**
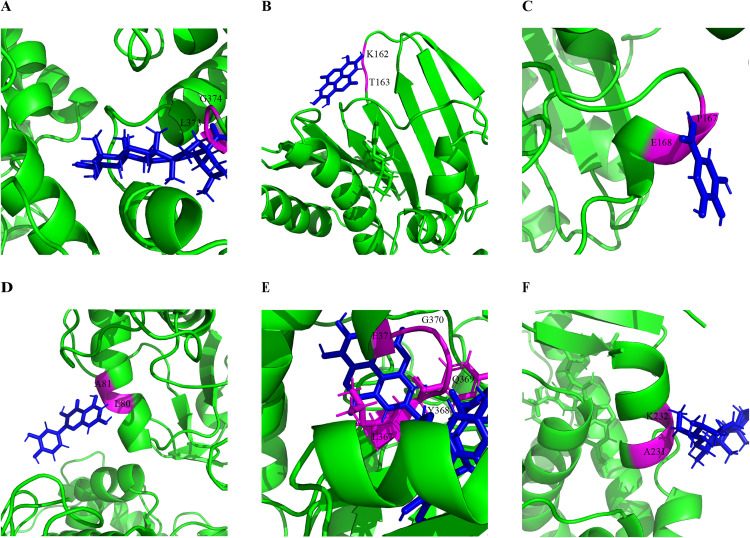
Molecular docking results of selected bioactive compounds from *L. speciosa* against target receptors/proteins. Docking simulations were conducted with SwissDock and analyzed using PyMOL to visualize binding interactions. (A) Corosolic acid docked to α-glucosidase (PDB ID: 3WY1); (B) Ellagic acid docked to DNA gyrase B (PDB ID: 6KZV); (C) Gallic acid docked to β-lactamase (PDB ID: 1XPB); (D) Quercetin docked to Peroxiredoxin 6 (PDB ID: 5B6M); (E) Lagerstroemin docked to α-glucosidase (PDB ID: 3WY1); and **(F)** γ-Sitosterol docked to PPAR-γ (PDB ID: 2P4Y). The images display the predicted binding modes and interaction sites (magenta) of each compound (blue) with its respective target protein (green), highlighting the interacting amino acids of the receptors.

Docking simulations revealed the interactions between major phytocompounds of *L. speciosa* and selected target proteins related to antidiabetic, antimicrobial, and antioxidant functions (**Table 6**). Six abundant compounds from *L. speciosa*: corosolic acid, ellagic acid, quercetin, γ-sitosterol, gallic acid, and lagerstroemin demonstrated potential pharmacological properties (**Fig 7**). The analysis generated key parameters including the Attracting Cavities (AC) score, binding free energy (SwissParam score), and root-mean-square deviation (RMSD) to assess docking accuracy and stability. Molecular docking predicted optimal binding conformations by identifying favorable interaction sites and visualizing them using PyMOL (**Fig 8**). Among the ligands, lagerstroemin exhibited the strongest binding affinity toward α-glucosidase (SwissParam score: −8.9076). It was observed to occupy the enzyme’s catalytic region, indicating a well-defined interaction. Similar to lagerstroemin, corosolic acid demonstrated a robust interaction with α-glucosidase (AC score: 32.249751; SwissParam score: −6.9352). γ-Sitosterol showed effective binding to PPAR-γ, aligning stably within its ligand-binding domain (SwissParam score: −7.4781). Docking successfully at the β-lactamase active site, gallic acid validated the enzyme’s microbial activity (SwissParam score: −6.0175). Through hydrogen bonding and hydrophobic contacts, ellagic acid established an interaction with DNA gyrase B. A favorable binding orientation for quercetin was observed within the active site of peroxiredoxin 6. The root-mean-square deviation (RMSD) values of the docked complexes ranged from 13.6882 (gallic acid) to 41.1486 (lagerstroemin).

**Table 6:** Docking scores of the identified compounds from L. speciosa against target receptors/proteins.

Ligand	Receptor/protein	Function	AC Score	SwissParam Score	RMSD
Corosolic acid	α-glucosidase	Antidiabetic	32.249751	−6.9352	16.1318
Ellagic acid	DNA gyrase B	Antimicrobial	54.991493	−6.4180	30.7511
Gallic acid	β-lactamase	Antimicrobial resistance	−11.146726	−6.0175	13.6882
Quercetin	Peroxiredoxin 6	Antioxidant	10.616882	−6.8687	23.8563
Lagerstroemin	α-glucosidase	Antidiabetic	294.741811	−8.9076	41.1486
γ-Sitosterol	PPAR-γ	Lipid regulation	13.225651	−7.4781	37.9405

*Note*. AC: Attracting Cavities, PPAR-γ: Peroxisome proliferator-activated receptor gamma, RMSD: Root-Mean-Square Deviation.

## Discussion

This study encompasses a detailed evaluation of the phytochemical composition, antioxidant activity, and bioactivity of *L. speciosa* leaf extracts. The findings highlight their potential for biomedical applications. Methanol, ethanol, acetone, and n-hexane were used as solvents to extract bioactive compounds from *L. speciosa* leaves. Methanol and ethanol extracts yielded the highest concentrations of total phenolics, flavonoids, and proanthocyanidins content [46,47]. This is attributed to their higher polarity indices compared to acetone and n-hexane. Phenolic and flavonoid compounds are more effectively extracted by polar solvents like methanol and ethanol. Methanol (5.1) has the highest polarity index and showed the best extraction efficiency. Ethanol (4.3) is safer and more eco-friendly, also performed well. In contrast, acetone (5.1) and n-hexane (0.1) showed lower extraction efficiencies due to their reduced capacity to solubilize hydrophilic compounds [48]. The presence of these bioactive compounds aligns with previous research indicating their role in managing oxidative stress. They are also linked to mitigating chronic diseases such as cardiovascular disorders, neurodegenerative conditions, and cancer [49–51]. Antioxidants play a crucial role in neutralizing free radicals, reducing disease risk, and preventing cellular damage [52]. The antioxidant activity of *L. speciosa* extracts was evaluated using IC_50_ values, representing the concentration needed to inhibit 50% of free radical activity. Methanol and ethanol extracts exhibited significant antioxidant activity, with IC_50_ values ranging from 64.19 to 241.59 µg/mL. These values are comparable to reference standard (ascorbic acid) and they had substantial antioxidant potential. They are consistent with previous reports on *L. speciosa* and other phenolic- and flavonoid-rich medicinal plants [53]. The biological relevance of these IC_50_ values is significant for therapeutic applications. However, translating these in vitro results to practical therapeutic doses requires further pharmacokinetic and bioavailability studies. The extracts’ potential to combat oxidative stress-related diseases warrants further exploration through in vivo models and clinical trials.

The antioxidant potential of *L. speciosa* extracts was evaluated using DPPH, ABTS, superoxide, and nitric oxide radical scavenging assays. Ethanol extracts demonstrated the highest DPPH scavenging activity (85.4 ± 0.46%) at a concentration of 500 µg/mL. Methanol derived extract showed unparalleled superoxide radical scavenging activity, with a concentration of 300 µg/mL achieving a remarkable inhibition (81.2 ± 0.6%). This suggests that the extracts contain rich flavonoid and phenolic compounds, which act as effective electron donors and scavengers of superoxide radicals. Superoxide radicals are linked to oxidative stress-related diseases and inflammation [54]. They are known to damage macromolecules, lipids, proteins, and DNA, thereby accelerating aging and disease progression. The observed scavenging activities emphasize *L. speciosa*’s potential as a natural antioxidant source. This potential can help mitigate oxidative stress and improve health [55]. We observed promising nitric oxide (NO) scavenging activity in *L. speciosa* extract. Ethanol extracts showed the highest inhibition (79.94 ± 0.34%) at a concentration of 300 µg/mL. NO serves as a biological messenger and vasodilator. However, excessive production can generate reactive nitrogen species (RNS) like peroxynitrite, which are toxic to tissues [56]. The ability of *L. speciosa* extracts to scavenge NO radicals indicates potential protective benefits. These benefits could extend to chronic inflammatory diseases and certain cancers associated with NO overproduction [57,58]. The brine shrimp lethality assay (BSLA) revealed moderate cytotoxicity in the methanol extract of *L. speciosa* (LC_50_ = 601.8 µg/mL). Based on the criteria established by Mayer et al. (1982), LC_50_ values below 1000 μg/mL in cytotoxicity screening are indicative of bioactivity, while values below 250 μg/mL denote high toxicity. The cytotoxicity of our experimental plant extracts (LC_50_ = 601.8 µg/mL) was significantly lower compared to that of Vincristine sulfate (LC_50_ = 0.91 μg/mL), as reported by Ullah et al. [59]. The LC_50_ of *L. speciosa* suggests the presence of bioactive compounds with anticancer potential. Compounds such as ellagic acid and gallic acid, previously isolated from *Lagerstroemia* species, have shown significant cytotoxicity in similar assays [60]. The moderate cytotoxicity indicates that *L. speciosa* may selectively target damaged cells over normal cells. Future studies are required to confirm selective cytotoxicity. These studies should use well-characterized human cancer cell lines, such as HeLa (cervical cancer) and HepG2 (hepatocellular carcinoma), alongside validated assays, including MTT, SRB, and Calcein assays [61]. These assays are widely used to evaluate cell viability and cytotoxicity. They provide reliable data on the selective toxicity of *L. speciosa* extracts toward cancer cells compared to normal cells. These approaches will aid in confirming the therapeutic efficacy and safety dose of the extracts. Additionally, apoptosis and cell cycle analysis using flow cytometry, combined with in vivo preclinical cancer models, are necessary to validate these findings. These steps will help elucidate the mechanisms of action and therapeutic applicability of *L. speciosa* extracts.

The antimicrobial activity of *L. speciosa* extracts is attributed to phenolic compounds, known for their inhibitory effects on microbial growth. Phenolic compounds in *L. speciosa* likely exert their antimicrobial effects through mechanisms such as disrupting microbial cell walls, inhibiting key enzymes, and chelating essential metal ions [62]. Specifically, Phenolic compounds containing a C3 side chain demonstrate antimicrobial properties, partly through their ability to decrease oxidative stress levels [63]. Moreover, they inhibit critical enzymes involved in microbial metabolic pathways, limiting nutrient acquisition and cellular functions necessary for survival [64]. The inhibition zones indicate moderate antimicrobial activity against bacterial and fungal strains (i.e., *Escherichia coli*, *Staphylococcus aureus*, *Candida albicans*, and *Saccharomyces cerevisiae*). These strains were chosen for their clinical significance as common pathogens. *Escherichia coli* and *Staphylococcus aureus* are key bacterial species associated with gastrointestinal and skin infections, respectively [65]. *Candida albicans* and *Saccharomyces cerevisiae* are well-documented fungal pathogens associated with opportunistic infections [66]. While the activity was lower than conventional antibiotics like ampicillin and antifungals like nystatin. The results suggest potential for complementary use in antimicrobial strategies, particularly in addressing antibiotic resistance. These results coincide with the previous studies that phenolic chemicals enhance their antibacterial activities [67]. Further studies could fully unlock its potential for clinical and commercial applications. Major steps like refining extraction methods, testing synergistic effects, and conducting in vivo studies are essential to evaluate efficacy and safety. *L. speciosa* extracts exhibited significant inhibition of α-amylase and α-glucosidase, key enzymes in carbohydrate metabolism. The methanol extract showed the highest α-glucosidase inhibitory activity, with an IC_50_ of 240.11 µg/mL, comparable to other natural inhibitors like *Camellia sinensis* and *Moringa oleifera* [68,69]. This dual inhibition of α-amylase and α-glucosidase suggests potential antidiabetic effects by delaying carbohydrate digestion and reducing postprandial glucose spikes. The inhibitory mechanism may involve multiple interactions between the bioactive compounds and enzyme structures. Polyphenols from *L. speciosa* may form hydrogen bonds with polar residues at the active sites of α-amylase and α-glucosidase, blocking substrates from binding [70]. Flavonoids, particularly those with hydroxyl groups at specific positions, can potentially form complexes with metal ions essential for enzyme function, such as the calcium required for α-amylase activity. Tannins, with their multiple phenolic hydroxyl groups, may induce conformational changes in the enzymes through hydrophobic interactions with aromatic amino acid residues surrounding the active site. Additionally, these phytochemicals might act as competitive inhibitors by mimicking the structure of natural substrates, or as non-competitive inhibitors by binding to allosteric sites that alter enzyme conformation and catalytic efficiency. The synergistic effect of multiple compounds present in the extract could also enhance inhibitory potency through simultaneous interactions at different binding sites, collectively modifying enzyme kinetics and substrate accessibility.

Our *in silico* analysis uncovered considerable pharmacological potential *for L. speciosa* phytochemicals against a diverse range of biological targets. PASS analysis indicated diverse bioactivities with high probability scores (Pa), specifically potential insulin-promoting, enzymatic inhibitory, and chemopreventive effects [71]. The strong affinities of corosolic acid and lagerstroemin for α-glucosidase support their potential as antidiabetic agents. In a similar fashion, quercetin displayed promising antioxidant activity, while ellagic acid notably exhibited antimicrobial activity [72]. These effects were supported by high docking scores and stable interactions with peroxiredoxin 6 and DNA gyrase B. Pharmacokinetic profiling indicated that ellagic acid, quercetin, and gallic acid satisfied Lipinski’s Rule of Five, having high gastrointestinal (GI) absorption and moderate bioavailability [73]. Despite minor deviations from these rules, corosolic acid maintained favorable oral absorption profiles. Toxicological predictions indicated that all compounds were non-carcinogenic and non-mutagenic. However, predicted toxicity to honeybees and aquatic species raised potential environmental concerns. The binding capabilities of these compounds with their respective target proteins were further validated through molecular docking. The RMSD values indicated varying conformational stabilities where gallic acid formed the most stable complex. These computational insights highlight the therapeutic potential of *L. speciosa* constituents and provide a strong basis for future experimental validation [74]. The findings of this study elucidate the antioxidant, antimicrobial, cytotoxic, and enzymatic inhibitory properties of *L. speciosa* leaf extracts, supporting their traditional medicinal uses. Further research should identify and characterize specific bioactive compounds (e.g., quercetin, kaempferol) and evaluate their synergistic effects. *In vivo* studies are crucial to assess pharmacokinetics, bioavailability, and safety, paving the way for therapeutic applications. The findings emphasize the potential of *L. speciosa* as a source of plant-based medicinal compounds. These compounds could address oxidative stress, metabolic imbalances, and infections. This aligns with the growing interest in natural products for managing global health challenges.

## Conclusions

This study highlights the therapeutic potential of *L. speciosa* leaf extracts, particularly methanol and ethanol extracts. These extracts are notably rich in key bioactive compounds, including corosolic acid, ellagic acid, gallic acid, quercetin, lagerstroemin, γ-sitosterol, campesterol, and phytol. Our findings reveal significant antioxidant activities through DPPH, ABTS, superoxide, and nitric oxide scavenging assays. Furthermore, the disk diffusion method confirmed their antimicrobial efficacy, demonstrating comparability to standard antibiotics. The brine shrimp toxicity assay also validated their cytotoxic potential, suggesting a promising role in anticancer therapeutics. Beyond these, the extracts exhibited potent α-amylase and α-glucosidase inhibitory activities indicating considerable antidiabetic potential. *In silico* ADME and molecular docking analysis provided compelling support for the diverse pharmacological activities observed with *L. speciosa*’s bioactive compounds. The *L. speciosa* leaf extract holds significant promise as a sustainable and effective natural resource for tackling pressing global health challenges.

## Supporting information

S1 Fig Standard curve of Gallic acid for determining total phenolic compounds. (PDF)

S2 Fig Catechin (CA) standard curve for determining total flavonoid content. (PDF)

S3 Fig Quercetin’s standard curve (QU) determination of total flavonol content. (PDF)

S4 Fig Standard curve of Catechin for the determination of proanthocyanidin. (PDF)

S1 File Extended Tables. (PDF)

## Abbreviations

*L. speciosa*: *Lagerstroemia speciosa*
GPx: Glutathione Peroxidase
TPC: Total Phenolic Content
GAE: Gallic Acid Equivalents
TFC: Total Flavonoid Content
CE: Catechin Equivalents
QE: Quercetin Equivalent
CFU: Colony Forming Units
SDA: Sabouraud Dextrose Agar
RNS: Reactive Nitrogen Species
MTT: 3-(4,5-dimethylthiazol-2-yl)-2,5-diphenyltetrazolium bromide
SRB: Sulforhodamine B
FCR: Folin Ciocalteu reagent
NBT: Nitro Blue Tetrazolium
DPPH: 2,2-Diphenyl-1-picrylhydrazyl
ABTS: 2,2′-Azino-bis (3-ethylbenzothiazoline-6-sulfonic acid)
DNSA 3: 5-Dinitrosalicylic Acid
C_50_: Half Maximal Inhibitory Concentration
BSLA: Brine Shrimp Lethality Assay
IZ: Inhibition Zone
MIC: Minimum Inhibitory Concentration
MBC: Minimum Bactericidal Concentration
MFC: Minimum Fungicidal Concentration
LC_50_: Lethal Concentration for 50% of the population
R^2^: Coefficient of Determination
NO: Nitric Oxide
SOD: Superoxide Dismutase
PBS: Phosphate Buffered Saline
DMSO: Dimethyl Sulfoxide
ANOVA: Analysis of Variance
SD: Standard Deviation
PPAR-γ: Peroxisome proliferator-activated receptor gamma
Pa: Probable activity
Pi: Probable inactivity
PASS: Prediction of Activity Spectra for Substances
LogP: Lipophilicity
SMILES: Simplified Molecular Input Line Entry System
ADME: Absorption, Distribution, Metabolism and Excretion
TPSA: Topological Polar Surface Area
AC: Attracting Cavities
AdmetSAR: Absorption, Distribution, Metabolism, Excretion, and Toxicity Structure-Activity Relationship.

## References

[1] WHO GS. Global status report on noncommunicable diseases 2014. 2014. https://apps.who.int/iris/bitstream/10665/148114/1/9789241564854_eng.pdf?ua=1

[2] Nediani C, Dinu M. Oxidative Stress and Inflammation as Targets for Novel Preventive and Therapeutic Approaches in Non-Communicable Diseases II. Antioxidants (Basel). 2022;11(5):824. doi: 10.3390/antiox11050824 35624688 PMC9137651

[3] Crisóstomo L, Oliveira PF, Alves MG. Antioxidants, Oxidative Stress, and Non-Communicable Diseases. Antioxidants (Basel). 2022;11(6):1080. doi: 10.3390/antiox11061080 35739977 PMC9220197

[4] Rahal A, Kumar A, Singh V, Yadav B, Tiwari R, Chakraborty S, et al. Oxidative stress, prooxidants, and antioxidants: the interplay. Biomed Res Int. 2014;2014:761264. doi: 10.1155/2014/761264 24587990 PMC3920909

[5] Ighodaro OM, Akinloye OA. First line defence antioxidants-superoxide dismutase (SOD), catalase (CAT) and glutathione peroxidase (GPX): Their fundamental role in the entire antioxidant defence grid. Alexandria Journal of Medicine. 2018;54(4):287–93. doi: 10.1016/j.ajme.2017.09.001

[6] Lobo V, Patil A, Phatak A, Chandra N. Free radicals, antioxidants and functional foods: Impact on human health. Pharmacogn Rev. 2010;4(8):118–26. doi: 10.4103/0973-7847.70902 22228951 PMC3249911

[7] Nostro A, Germanò MP, D’angelo V, Marino A, Cannatelli MA. Extraction methods and bioautography for evaluation of medicinal plant antimicrobial activity. Lett Appl Microbiol. 2000;30(5):379–84. doi: 10.1046/j.1472-765x.2000.00731.x 10792667

[8] Mancuso G, Midiri A, Gerace E, Biondo C. Bacterial Antibiotic Resistance: The Most Critical Pathogens. Pathogens. 2021;10(10):1310. doi: 10.3390/pathogens10101310 34684258 PMC8541462

[9] Buckland D. Antimicrobial resistance and the race to find new antibiotics. Prescriber. 2017;28(1):12–5. doi: 10.1002/psb.1528

[10] Sharma SB, Gupta R. Drug development from natural resource: a systematic approach. Mini Rev Med Chem. 2015;15(1):52–7. doi: 10.2174/138955751501150224160518 25986040

[11] Khazir J, Mir BA, Mir SA, Cowan D. Natural products as lead compounds in drug discovery. J Asian Natural Products Research. 2013;15(7):764–88. doi: 10.1080/10286020.2013.798314

[12] Imming P. Molecular targets of natural drug substances: idiosyncrasies and preferences. Planta Med. 2010;76(16):1794–801. doi: 10.1055/s-0030-1250236 20717868

[13] Choudhary S, Singh PK, Verma H, Singh H, Silakari O. Success stories of natural product-based hybrid molecules for multi-factorial diseases. Eur J Med Chem. 2018;151:62–97. doi: 10.1016/j.ejmech.2018.03.057 29605809

[14] He L, He T, Farrar S, Ji L, Liu T, Ma X. Antioxidants maintain cellular redox homeostasis by elimination of reactive oxygen species. Cell Physiol Biochem. 2017;44(2):532–53. doi: 10.1159/000485089 29145191

[15] Mirończuk-Chodakowska I, Witkowska AM, Zujko ME. Endogenous non-enzymatic antioxidants in the human body. Adv Med Sci. 2018;63(1):68–78. doi: 10.1016/j.advms.2017.05.005 28822266

[16] Ma L, Zhang M, Zhao R, Wang D, Ma Y, Li A. Plant natural products: promising resources for cancer chemoprevention. Molecules. 2021;26(4):933. doi: 10.3390/molecules26040933 33578780 PMC7916513

[17] Liu F, Kim J, Li Y, Liu X, Li J, Chen X. An extract of lagerstroemia speciosa L. has insulin-like glucose uptake–stimulatory and adipocyte differentiation–inhibitory activities in 3T3-L1 Cells. J Nutrition. 2001;131(9):2242–7. doi: 10.1093/jn/131.9.2242 11533261

[18] Sharmin T, Rahman MS, Mohammadi H. Investigation of biological activities of the flowers of Lagerstroemia speciosa, the Jarul flower of Bangladesh. BMC Complement Altern Med. 2018;18(1):231. doi: 10.1186/s12906-018-2286-6 30081877 PMC6080514

[19] Tripathi SK, Behera S, Panda M, Zengin G, Biswal BK. A comprehensive review on pharmacology and toxicology of bioactive compounds of lagerstroemia speciosa(L.) Pers. CTM. 2021;7(4):504–13. doi: 10.2174/2215083806999201211213931

[20] Stohs SJ, Miller H, Kaats GR. A review of the efficacy and safety of banaba (Lagerstroemia speciosa L.) and corosolic acid. Phytother Res. 2012;26(3):317–24. doi: 10.1002/ptr.3664 22095937

[21] Hussain F, Ganguly A, Hossain MS, Rahman SA. Analgesic and anti-diarrhoeal activities of Lagerstroemia speciosa roots in experimental animal model. Dhaka Univ J Pharm Sci. 2015;13(1):57–62. doi: 10.3329/dujps.v13i1.21860

[22] Samananda Singh L, Singh WS. Multifaceted therapeutic potential of corosolic acid: A novel bioactive compound. Obesity Medicine. 2024;49:100548. doi: 10.1016/j.obmed.2024.100548

[23] Yamada K, Hosokawa M, Fujimoto S, Fujiwara H, Fujita Y, Harada N, et al. Effect of corosolic acid on gluconeogenesis in rat liver. Diabetes Res Clin Pract. 2008;80(1):48–55. doi: 10.1016/j.diabres.2007.11.011 18177973

[24] Rohit Singh T, Ezhilarasan D. Lagerstroemia speciosa (L.) Pers., ethanolic leaves extract attenuates dapsone-induced liver inflammation in rats. Drug Chem Toxicol. 2022;45(5):2361–70. doi: 10.1080/01480545.2021.1945079 34225555

[25] Pal LC, kumar A, Pande V, Ch.V. Rao R. Hepatoprotective effect of bioactive fraction of lagerstroemia speciosa (l.) pers. bark against monosodium glutamate-induced liver toxicity. PJ. 2020;12(6s):1630–40. doi: 10.5530/pj.2020.12.223

[26] Basha MP, Saumya SM. Influence of fluoride on streptozotocin induced diabetic nephrotoxicity in mice: protective role of Asian ginseng (Panax ginseng) & banaba (Lagerstroemia speciosa) on mitochondrial oxidative stress. Indian J Med Res. 2013;137(2):370–9. 23563382 PMC3657862

[27] Unno T, Sugimoto A, Kakuda T. Xanthine oxidase inhibitors from the leaves of Lagerstroemia speciosa (L.) Pers. J Ethnopharmacol. 2004;93(2–3):391–5. doi: 10.1016/j.jep.2004.04.012 15234783

[28] Khunnawattanakul W, Boonma P, Kampetch R, Jaruchotikamol A, Cushnie B, Rattanakiat S, et al. Inhibitory actions of lagerstroemia speciosa (l.) pers. aqueous and ethanolic leaf extracts against carbohydrate-digesting enzymes. PJ. 2018;10(6s):s113–8. doi: 10.5530/pj.2018.6s.22

[29] Sirikhansaeng P, Tanee T, Sudmoon R, Chaveerach A. Major phytochemical as γ-sitosterol disclosing and toxicity testing in lagerstroemia species. Evid Based Complement Alternat Med. 2017;2017:7209851. doi: 10.1155/2017/7209851 28191023 PMC5278189

[30] Alkahtani S, Hasnain MS, Algamdy H, Aljarba NH, AlKahtane A. Acute and sub-acute oral toxicity Lagerstroemia speciosa in Sprague-Dawley rats. Saudi J Biol Sci. 2022;29(3):1585–91. doi: 10.1016/j.sjbs.2021.11.005 35280577 PMC8913382

[31] Singleton VL, Orthofer R, Lamuela-Raventós RM. [14] Analysis of total phenols and other oxidation substrates and antioxidants by means of folin-ciocalteu reagent. Methods in Enzymology. Elsevier. 1999. p. 152–78. doi: 10.1016/s0076-6879(99)99017-1

[32] Chang C-C, Yang M-H, Wen H-M, Chern J-C. Estimation of total flavonoid content in propolis by two complementary colometric methods. Journal of Food and Drug Analysis. 2020;10(3). doi: 10.38212/2224-6614.2748

[33] Kumaran A, Joel Karunakaran R. In vitro antioxidant activities of methanol extracts of five Phyllanthus species from India. LWT - Food Science and Technology. 2007;40(2):344–52. doi: 10.1016/j.lwt.2005.09.011

[34] Sun B, Ricardo-da-Silva JM, Spranger I. Critical factors of vanillin assay for catechins and proanthocyanidins. J Agric Food Chem. 1998;46(10):4267–74. doi: 10.1021/jf980366j

[35] Brand-Williams W, Cuvelier ME, Berset C. Use of a free radical method to evaluate antioxidant activity. LWT - Food Science and Technology. 1995;28(1):25–30. doi: 10.1016/s0023-6438(95)80008-5

[36] Re R, Pellegrini N, Proteggente A, Pannala A, Yang M, Rice-Evans C. Antioxidant activity applying an improved ABTS radical cation decolorization assay. Free Radic Biol Med. 1999;26(9–10):1231–7. doi: 10.1016/s0891-5849(98)00315-3 10381194

[37] Beauchamp C, Fridovich I. Superoxide dismutase: improved assays and an assay applicable to acrylamide gels. Anal Biochem. 1971;44(1):276–87. doi: 10.1016/0003-2697(71)90370-8 4943714

[38] Martinez CA, Loureiro ME, Oliva MA, Maestri M. Differential responses of superoxide dismutase in freezing resistant Solanum curtilobum and freezing sensitive Solanum tuberosum subjected to oxidative and water stress. Plant Sci. 2001;160(3):505–15. doi: 10.1016/s0168-9452(00)00418-0 11166438

[39] Sreejayan, Rao MN. Nitric oxide scavenging by curcuminoids. J Pharm Pharmacol. 1997;49(1):105–7. doi: 10.1111/j.2042-7158.1997.tb06761.x 9120760

[40] Meyer BN, Ferrigni NR, Putnam JE, Jacobsen LB, Nichols DE, McLaughlin JL. Brine shrimp: a convenient general bioassay for active plant constituents. Planta Med. 1982;45(5):31–4. doi: 10.1055/s-2007-971236 17396775

[41] Bauer AW, Kirby WMM, Sherris JC, Turck M. Antibiotic susceptibility testing by a standardized single disk method. American J Clinical Pathol. 1966;45(4_ts):493–6. doi: 10.1093/ajcp/45.4_ts.493 5325707

[42] Mbaveng AT, Ngameni B, Kuete V, Simo IK, Ambassa P, Roy R, et al. Antimicrobial activity of the crude extracts and five flavonoids from the twigs of Dorstenia barteri (Moraceae). J Ethnopharmacol. 2008;116(3):483–9. doi: 10.1016/j.jep.2007.12.017 18280679

[43] Miller GL. Use of dinitrosalicylic acid reagent for determination of reducing sugar. Anal Chem. 1959;31(3):426–8. doi: 10.1021/ac60147a030

[44] Kazeem MI, Adamson JO, Ogunwande IA. Modes of inhibition of α -amylase and α -glucosidase by aqueous extract of Morinda lucida Benth leaf. Biomed Res Int. 2013;2013:527570. doi: 10.1155/2013/527570 24455701 PMC3884628

[45] Berman HM, Westbrook J, Feng Z, Gilliland G, Bhat TN, Weissig H, et al. The protein data bank. Nucleic Acids Res. 2000;28(1):235–42. doi: 10.1093/nar/28.1.235 10592235 PMC102472

[46] Sulaiman SF, Sajak AAB, Ooi KL, Supriatno, Seow EM. Effect of solvents in extracting polyphenols and antioxidants of selected raw vegetables. J Food Composition Anal. 2011;24(4–5):506–15. doi: 10.1016/j.jfca.2011.01.020

[47] Ali H, Hasi RY, Islam M, Haque MS, Alkhanani MF, Almalki AH, et al. Antioxidant, cytotoxic and apoptotic activities of the rhizome of Zingiber zerumbet Linn. in Ehrlich ascites carcinoma bearing Swiss albino mice. Sci Rep. 2022;12(1):12150. doi: 10.1038/s41598-022-15498-8 35840634 PMC9287333

[48] Kajdzanoska M, Petreska J, Stefova M. Comparison of different extraction solvent mixtures for characterization of phenolic compounds in strawberries. J Agric Food Chem. 2011;59(10):5272–8. doi: 10.1021/jf2007826 21495681

[49] Rudrapal M, Khairnar SJ, Khan J, Dukhyil AB, Ansari MA, Alomary MN, et al. Dietary polyphenols and their role in oxidative stress-induced human diseases: insights into protective effects, antioxidant potentials and mechanism(s) of action. Front Pharmacol. 2022;13:806470. doi: 10.3389/fphar.2022.806470 35237163 PMC8882865

[50] Mutha RE, Tatiya AU, Surana SJ. Flavonoids as natural phenolic compounds and their role in therapeutics: an overview. Futur J Pharm Sci. 2021;7(1):25. doi: 10.1186/s43094-020-00161-8 33495733 PMC7816146

[51] Asaduzzaman M, Nahar L, Hasan M, Khatun A, Shajedul HM. Antihyperglycemic Activity, antihyperlipedemic activity, hepatoprotective activity and histopathological analysis of natural honey in streptozotocin induced diabetic rats. J Cytol Histol. 2016;7: 402. doi: 10.4172/2157-7099.1000402

[52] Pisoschi AM, Pop A. The role of antioxidants in the chemistry of oxidative stress: a review. Eur J Med Chem. 2015;97:55–74. doi: 10.1016/j.ejmech.2015.04.040 25942353

[53] Junaid S, K N. Rakesh KNR, Dileep N, Poornima G, Kekuda TRP, Mukunda S. Total Phenolic Content and Antioxidant Activity of Seed Extract of Lagerstroemia Speciosa L. Chem Sci Trans. 2012;2(1):75–80. doi: 10.7598/cst2012.310

[54] Rastogi S, Pandey MM, Rawat AKS. Traditional herbs: a remedy for cardiovascular disorders. Phytomedicine. 2016;23(11):1082–9. doi: 10.1016/j.phymed.2015.10.012 26656228

[55] Unno T, Sakane I, Masumizu T, Kohno M, Kakuda T. Antioxidative activity of water extracts of lagerstroemia speciosa leaves. Biosci Biotechnol Biochem. 1997;61(10):1772–4. doi: 10.1271/bbb.61.1772 27393177

[56] Liaudet L, Soriano FG, Szabó C. Biology of nitric oxide signaling. Crit Care Med. 2000;28(4 Suppl):N37-52. doi: 10.1097/00003246-200004001-00005 10807315

[57] Pavithran S, Sujatha PS. Evaluation of anti-inflammatory properties of ethanolic leaf, flower and seed extracts of lagerstroemia speciosa (l.) pers (lythraceae) against carrageenan-induced acute inflammation in albino rats. JASR. 2022;13(09):48–53. doi: 10.55218/jasr.202213907

[58] Mousa AM, El-Sammad NM, Abdel-Halim AH, Anwar N, Khalil WKB, Nawwar M, et al. Lagerstroemia speciosa (L.) Pers Leaf Extract Attenuates Lung Tumorigenesis via Alleviating Oxidative Stress, Inflammation and Apoptosis. Biomolecules. 2019;9(12):871. doi: 10.3390/biom9120871 31842482 PMC6995620

[59] Ullah MO, Haque M, Urmi KF, Zulfiker AHM, Anita ES, Begum M, et al. Anti-bacterial activity and brine shrimp lethality bioassay of methanolic extracts of fourteen different edible vegetables from Bangladesh. Asian Pac J Trop Biomed. 2013;3(1):1–7. doi: 10.1016/S2221-1691(13)60015-5 23570009 PMC3609385

[60] Rohit Singh T, Ezhilarasan D. Ethanolic extract of lagerstroemia speciosa (L.) pers., induces apoptosis and cell cycle arrest in HepG2 cells. Nutr Cancer. 2020;72(1):146–56. doi: 10.1080/01635581.2019.1616780 31149840

[61] Mueller H, Kassack MU, Wiese M. Comparison of the usefulness of the MTT, ATP, and calcein assays to predict the potency of cytotoxic agents in various human cancer cell lines. J Biomol Screen. 2004;9(6):506–15. doi: 10.1177/1087057104265386 15452337

[62] Jiamboonsri P, Eurtivong C, Wanwong S. Assessing the potential of gallic acid and methyl gallate to enhance the efficacy of β-lactam antibiotics against methicillin-resistant staphylococcus aureus by targeting β-lactamase: in silico and in vitro studies. Antibiotics (Basel). 2023;12(11):1622. doi: 10.3390/antibiotics12111622 37998824 PMC10669207

[63] Baba SA, Malik SA. Determination of total phenolic and flavonoid content, antimicrobial and antioxidant activity of a root extract of Arisaema jacquemontii Blume. J Taibah University for Sci. 2015;9(4):449–54. doi: 10.1016/j.jtusci.2014.11.001

[64] Zlitni S, Ferruccio LF, Brown ED. Metabolic suppression identifies new antibacterial inhibitors under nutrient limitation. Nat Chem Biol. 2013;9(12):796–804. doi: 10.1038/nchembio.1361 24121552 PMC3970981

[65] Otto M. Staphylococci in the human microbiome: the role of host and interbacterial interactions. Curr Opin Microbiol. 2020;53:71–7. doi: 10.1016/j.mib.2020.03.003 32298967

[66] Liu Z, MacAlpine J, Robbins N, Cowen LE. Construction of Candida albicans Strains with ATP-Analog-Sensitive Protein Kinase A and Hog1. mSphere. 2023;8(3):e0009523. doi: 10.1128/msphere.00095-23 37039635 PMC10286698

[67] Sinelius S, Lady J, Yunardy M, Tjoa E, Nurcahyanti ADR. Antibacterial activity of Lagerstreomia speciosa and its active compound, corosolic acid, enhances cefotaxime inhibitory activity against Staphylococcus aureus. J Appl Microbiol. 2023;134(8):lxad171. doi: 10.1093/jambio/lxad171 37541956

[68] Bhatia A, Singh B, Arora R, Arora S. In vitro evaluation of the α-glucosidase inhibitory potential of methanolic extracts of traditionally used antidiabetic plants. BMC Complement Altern Med. 2019;19(1):74. doi: 10.1186/s12906-019-2482-z 30909900 PMC6434821

[69] Magaji UF, Sacan O, Yanardag R. Alpha amylase, alpha glucosidase and glycation inhibitory activity of Moringa oleifera extracts. South African Journal of Botany. 2020;128:225–30. doi: 10.1016/j.sajb.2019.11.024

[70] Proença C, Ribeiro D, Freitas M, Fernandes E. Flavonoids as potential agents in the management of type 2 diabetes through the modulation of α-amylase and α-glucosidase activity: a review. Crit Rev Food Sci Nutr. 2022;62(12):3137–207. doi: 10.1080/10408398.2020.1862755 33427491

[71] Filimonov DA, Lagunin AA, Gloriozova TA, Rudik AV, Druzhilovskii DS, Pogodin PV, et al. Prediction of the biological activity spectra of organic compounds using the pass online web resource. Chem Heterocycl Comp. 2014;50(3):444–57. doi: 10.1007/s10593-014-1496-1

[72] Al-Nour MY, Ibrahim MM, Elsaman T. Ellagic acid, kaempferol, and quercetin from acacia nilotica: promising combined drug with multiple mechanisms of action. Curr Pharmacol Rep. 2019;5(4):255–80. doi: 10.1007/s40495-019-00181-w 32226726 PMC7100491

[73] Pais ACS, Coscueta ER, Pintado MM, Silvestre AJD, Santos SAO. Exploring the bioaccessibility and intestinal absorption of major classes of pure phenolic compounds using in vitro simulated gastrointestinal digestion. Heliyon. 2024;10(7):e28894. doi: 10.1016/j.heliyon.2024.e28894 PMC11016601 38623258

[74] Raju L, Lipin R, Eswaran R. Identification, ADMET evaluation and molecular docking analysis of Phytosterols from Banaba (Lagerstroemia speciosa (L.)Pers) seed extract against breast cancer. In Silico Pharmacol. 2021;9(1):43. doi: 10.1007/s40203-021-00104-y 34367875 PMC8289922

