# Supplementary figures and images for: Integrating bioactivity and molecular simulations to explore the pharmacological landscape of Lagerstroemia speciosa leaf extract
Source: PLoS One. 2025 Dec 29;20(12):e0339566. doi: 10.1371/journal.pone.0339566 (PMC12747374; doi:10.1371/journal.pone.0339566)

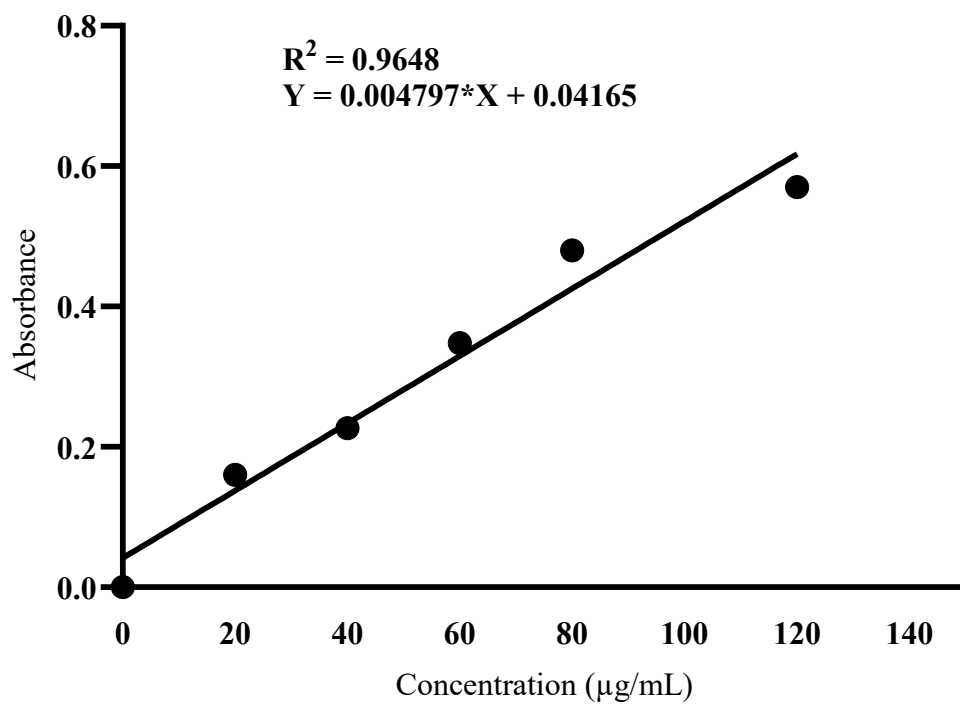

**S1 Fig. Standard curve of Gallic acid for determining total phenolic compounds.**

Supplement: S1 Fig — (PDF) [file pone.0339566.s001.pdf]

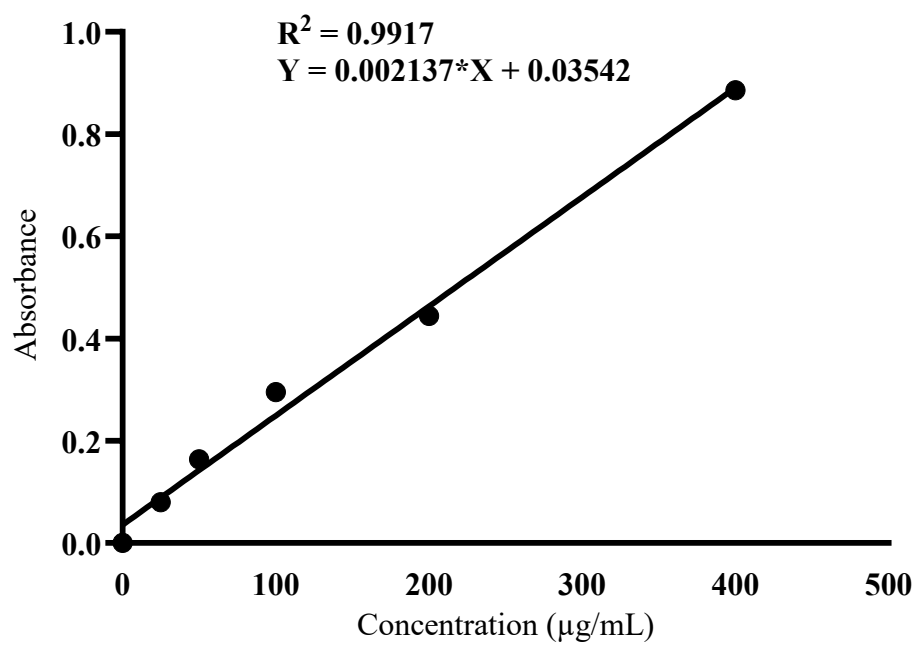

**S2 Fig. Catechin (CA) standard curve for determining total flavonoid content.**

Supplement: S2 Fig — (PDF) [file pone.0339566.s002.pdf]

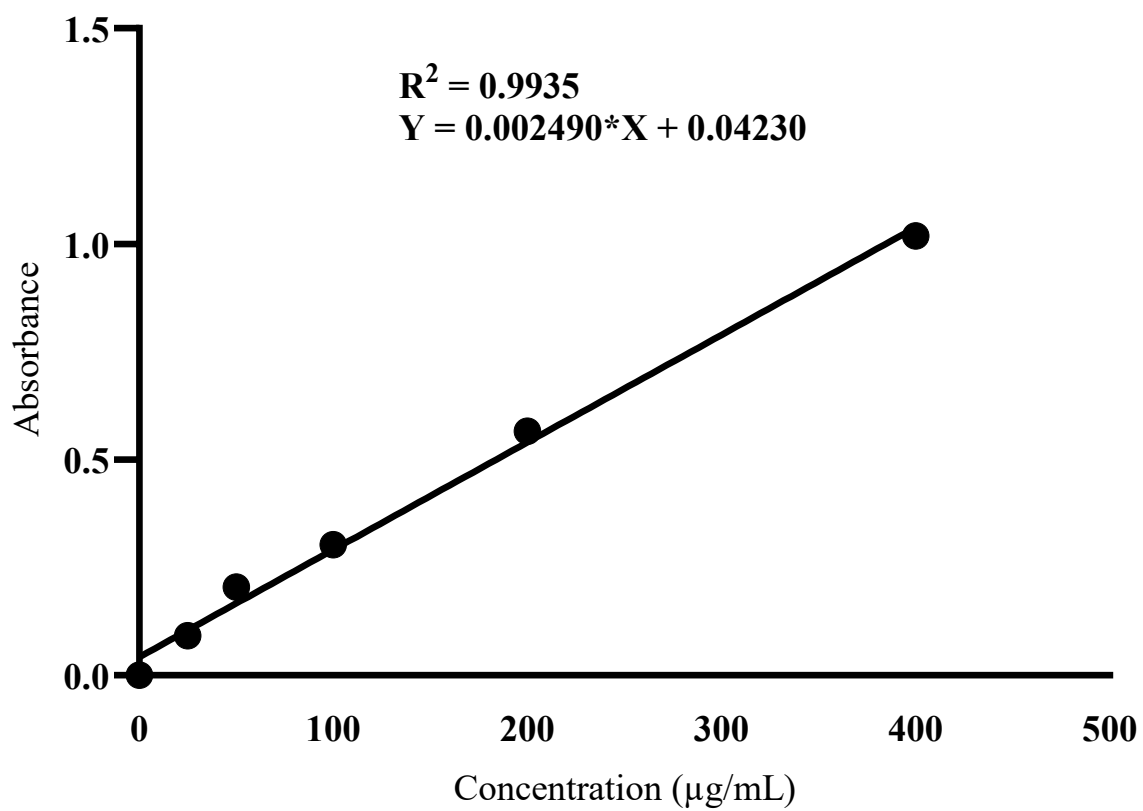

**S3 Fig. Quercetin's standard curve (QU) determination of total flavonol content.**

Supplement: S3 Fig — (PDF) [file pone.0339566.s003.pdf]

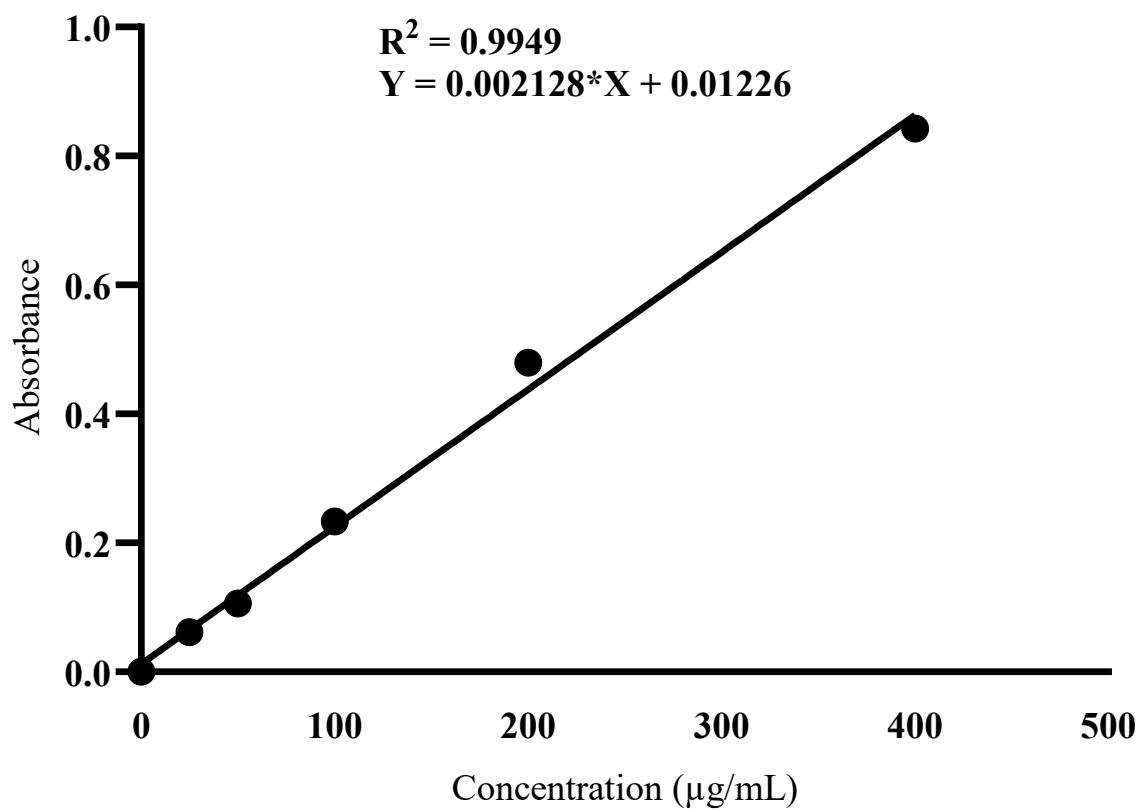

**S4 Fig. Standard curve of Catechin for the determination of proanthocyanidin.**

Supplement: S4 Fig — (PDF) [file pone.0339566.s004.pdf]
