# Supplementary material for: Integrating bioactivity and molecular simulations to explore the pharmacological landscape of Lagerstroemia speciosa leaf extract
Source: PLoS One. 2025 Dec 29;20(12):e0339566. doi: 10.1371/journal.pone.0339566 (PMC12747374; doi:10.1371/journal.pone.0339566)
Supplement: S1 File — (PDF) [file pone.0339566.s005.pdf]

S1 Table. Absorbance of Gallic acid (standard) at different concentrations.

Concentration (μg/mL)	Absorbance			Mean ± SD
	A	B	C	
20	0.158	0.16	0.163	0.160333 ± 0.0025
40	0.225	0.227	0.228	0.226667 ± 0.0015
60	0.348	0.345	0.35	0.347667 ± 0.0025
80	0.478	0.482	0.48	0.48 ± 0.002
120	0.568	0.57	0.573	0.570333 ± 0.0025

Note. **A**, **B**, and **C** represent three independent experiments. **SD** denotes “Standard deviation”.

S2 Table. Absorbance of Catechin (standard) at different concentrations.

Concentration (µg/mL)	Absorbance			Mean ± SD
	A	B	C	
25	0.08	0.078	0.081	0.080 ± 0.0015
50	0.165	0.167	0.160	0.164 ± 0.0036
100	0.291	0.296	0.298	0.295 ± 0.0036
200	0.442	0.444	0.446	0.444 ± 0.002
400	0.883	0.886	0.889	0.886 ± 0.003

Note. **A**, **B**, and **C** represent three independent experiments. **SD** denotes "Standard deviation".

S3 Table. Absorbance of Quercetin (QU) at different concentrations.

Concentration (μg/mL)	Absorbance			Mean ± SD
	A	B	C	
25	0.092	0.088	0.095	0.09167 ± 0.0035
50	0.203	0.206	0.204	0.20433 ± 0.0015
100	0.301	0.306	0.302	0.303 ± 0.0026
200	0.564	0.568	0.565	0.56567 ± 0.0021
400	1.018	1.022	1.016	1.01867 ± 0.0031

Note. **A**, **B**, and **C** represent three independent experiments. **SD** denotes "Standard deviation".

S4 Table. Absorbance of Catechin (standard) at different concentrations.

Concentration (µg/mL)	Absorbance			Mean ± SD
	A	B	C	
25	0.062	0.06	0.063	0.061667± 0.0015
50	0.105	0.11	0.103	0.106 ± 0.0036
100	0.23	0.237	0.232	0.233 ± 0.0036
200	0.479	0.482	0.478	0.479667 ± 0.0021
400	0.845	0.842	0.84	0.842333 ± 0.0025

Note. **A**, **B**, and **C** represent three independent experiments. **SD** denotes "Standard deviation".

Results of Phytochemical Screening of *L. speciosa* leaves extracts

The preliminary phytochemical screening tests revealed the presence of local plant extracts of alkaloid, carbohydrate, flavonoid, glycoside, resin, saponin, and steroids.

Extra Table 1. Phytochemical screening of *L. speciosa* leaves extracts

Name of tests		Name of sample extract			
		Methanol	Ethanol	Acetone	n-Hexane
Alkaloids	Dragendorff's test	+	+	+	+
	Hager's test	+	+	-	+
	Wagner's test	+	+	+	-
	Mayer's test	+	-	+	+
Carbohydrate test	Anthrone's test	+	+	+	+
	Benedict's test	+	+	+	+
	Fehling's test	+	+	+	+
	Molisch's test	+	+	-	+
Flavonoid test		+	+	+	+
Glycosides		+	+	-	+
Triterpenoids		+	+	-	-
Resins		+	+	+	+
Saponins		+	-	+	+
Steroids	Liebermann-Burchard's test	+	+	+	+
	Salkowski reaction	-	+	-	-
Tannins		+	+	+	+
Coumarins test		-	+	+	+
Cardio glycoside test		+	+	-	+
Anthracenosides test		-	+	+	+

Note. '+' denotes positive and '-' denotes negative in test here.

Results of phytochemical *Lagerstroemia speciosa* Extracts

Total Phenolic Content (TPC)

Each of the four *L. speciosa* extracts was analyzed for total phenolic content (TPC). The calibration curve for gallic acid demonstrated linearity within the concentration range of 20 - 120 µg/mL, with a correlation coefficient (R^2) of 0.9648 and a linear regression equation of **y = 0.004797x + 0.04165**, where **y** represents absorbance, and **x** denotes concentration (µg/mL). The TPC of *L. speciosa* leaf extracts (**Table 1.** and **Fig 1A.**) was calculated using the following formula:

Concentration, x = (y - 0.04165) / 0.004797 µg/mL.

Table 1. Total phenolic content of *L. speciosa* leaves extracts.

Name of extract	No. of sample	Absorbance	mg of Gallic acid / g of dried extract	mg of GAE ± SD
	1	0.274	48.50	
Methanol	2	0.275	48.68	48.80 ± 0.37
	3	0.278	49.22	
	1	0.261	45.83	
Ethanol	2	0.265	46.54	46.18 ± 0.35
	3	0.263	46.18	
	1	0.191	31.18	
Acetone	2	0.193	31.54	31.60 ± 0.45
	3	0.196	32.08	
	1	0.097	11.48	
n-Hexane	2	0.097	11.54	11.60 ± 0.45
	3	0.100	12.08	

Note. **GAE** and **SD** denote “Gallic acid equivalent” and “Standard deviation”, respectively.

Total Flavonoid Content (TFC)

The total flavonoid content (TFC) in the four extracts of *L. speciosa* was determined using a calibration curve for catechin (CA), which exhibited linearity within the concentration range of 25 - 400 µg/mL. The calibration curve yielded a correlation coefficient (R^2) of 0.9917 and a linear regression equation of **y = 0.002137x + 0.03542**, where **y** represents absorbance and **x** denotes concentration (µg/mL). The TFC of *L. speciosa* leaf extracts (**Table 2.** and **Fig 1B.**) was calculated using the equation:

Concentration, x = (y - 0.03542) / 0.002137 µg/mL.

Table 2. Total Flavonoid content of *L. speciosa* leaves extracts.

Name of extract	No. of sample	Absorbance	mg of Catechin / g of dried extract	mg of Catechin ± SD
	1	0.053	8.216	
Methanol	2	0.051	7.354	8.0843 ± 0.67
	3	0.054	8.683	
	1	0.055	9.309	
Ethanol	2	0.053	8.45	9.181 ± 0.67
	3	0.056	9.784	
	1	0.048	5.673	
Acetone	2	0.046	4.918	5.555 ± 0.59
	3	0.048	6.074	
	1	0.052	7.686	
n-Hexane	2	0.049	6.553	7.5043 ± 0.87
	3	0.053	8.274	

Note. **CA** and **SD** denote "Catechin" and "Standard deviation", respectively.

Total Flavonol content

The calibration curve for quercetin (QU) demonstrated linearity within the concentration range of 25 - 400 µg/mL, with a correlation coefficient (R^2) of 0.9935 and a linear regression equation of **y = 0.002490x + 0.04230**, where **y** represents absorbance and **x** denotes concentration (µg/mL). The total flavonol content of *L. speciosa* leaf extracts (**Table 3.** and **Fig 2A.**) was calculated using the equation:

Concentration, x = (y - 0.04230) / 0.002490 µg/mL.

Table 3. Total flavonol content of *L. speciosa* leaves extracts.

Name of extract	No. of sample	Absorbance	mg of quercetin / g of dried extract	mg of quercetin ± SD
	1	0.062	7.764	
Methanol	2	0.059	6.888	7.6787 ± 0.752
	3	0.063	8.384	
	1	0.064	8.601	
Ethanol	2	0.061	7.626	8.4513 ± 0.762
	3	0.065	9.127	
	1	0.053	4.183	
Acetone	2	0.051	3.446	4.0587 ± 0.561
	3	0.054	4.547	
	1	0.056	5.384	
n-Hexane	2	0.054	4.637	5.255 ± 0.564
	3	0.057	5.744	

Note. **SD** denotes “Standard deviation”.

Total proanthocyanidin content

The calibration curve for catechin exhibited linearity within the concentration range of 25 - 400 µg/mL, with a correlation coefficient (R^2) of 0.9949 and a linear regression equation of **y = 0.002128x + 0.01226**, where **y** represents absorbance and **x** denotes concentration (µg/mL). The total proanthocyanidin content of *L. speciosa* leaf extracts (**Table 4.** and **Fig 2B.**)
was calculated using the equation:

Concentration, x = (y - 0.01226) / 0.002128 µg/mL.

Table 4. Total Proanthocyanidin content of *L. speciosa* leaves extracts.

Name of extract	No. of sample	Absorbance	mg of Catechin / g of dried extract	mg of Catechin ± SD
	1	0.073	28.77	
Methanol	2	0.075	29.69	29.54 ± 0.71
	3	0.076	30.16	
	1	0.089	35.97	
Ethanol	2	0.084	33.74	34.80 ± 1.12
	3	0.086	34.69	
	1	0.042	13.97	
Acetone	2	0.044	14.69	14.60 ± 0.60
	3	0.045	15.16	
	1	0.051	18.02	
n-Hexane	2	0.053	18.97	18.74 ± 0.63
	3	0.053	19.22	

Note. **SD** denotes “Standard deviation”.

DPPH free radical scavenging activity

The DPPH free radical scavenging assay results by *L. speciosa* leaves extracts are shown in **Table 5. & Fig 3A.** The DPPH scavenging activity of four extracts were compared to reference standard (Ascorbic acid).

Table 5. The DPPH free radical scavenging activity of *L. speciosa* leaves extracts is followed.

Conc.(µg/mL)	Extract	% of inhibitions			Mean ± SD
		1	2	3	
20	Methanol	16.37	17.3	17.81	17.16 ± 0.79
	Ethanol	14.87	15.02	16.04	15.31 ± 0.64
	Acetone	24.36	24.55	25.16	24.69 ± 0.41
	n-Hexane	10.22	10.1	10.7	10.34 ± 0.32
	Ascorbic acid	17.88	17.82	18.96	18.22 ± 0.64
40	Methanol	31.5	32.32	32.75	32.19 ± 0.64
	Ethanol	33.62	33.75	34.33	33.9 ± 0.38
	Acetone	33.7	33.92	34.44	34.02 ± 0.31
	n-Hexane	23.19	23.27	24.34	23.6 ± 0.62
	Ascorbic acid	32.61	33.62	33.76	33.33 ± 0.63
80	Methanol	48.79	49.21	50.05	49.35 ± 0.64
	Ethanol	55.52	55.77	56.56	55.95 ± 0.54
	Acetone	45.98	46.84	47.85	46.89 ± 0.94
	n-Hexane	33.57	33.8	34.48	33.95 ± 0.47
	Ascorbic acid	49.29	49.55	49.99	49.61 ± 0.35
160	Methanol	63.76	64.15	64.66	64.19 ± 0.45
	Ethanol	67.79	68.2	68.79	68.26 ± 0.50
	Acetone	71.78	72.11	72.62	72.17 ± 0.42
	n-Hexane	40.9	41.26	41.8	41.32 ± 0.45
	Ascorbic acid	80.18	80.29	80.61	80.36 ± 0.22

	Methanol	74.03	74.19	74.5	74.24 ± 0.23
	Ethanol	81.52	82.24	82.78	82.18 ± 0.63
**320**	Acetone	76	76.2	76.52	76.24 ± 0.26
	n-Hexane	51.45	51.75	52.23	51.81 ± 0.39
	Ascorbic acid	90.22	91.2	92.33	91.25 ± 1.06
	Methanol	81.98	82.44	82.9	82.44 ± 0.46
	Ethanol	85.01	85.29	85.9	85.4 ± 0.46
**500**	Acetone	78.88	79.19	79.8	79.29 ± 0.47
	n-Hexane	64.57	65.25	65.81	65.21 ± 0.62
	Ascorbic acid	94.7	95.2	95.91	95.27 ± 0.61

Note. **1, 2,** and **3** represent three independent experiments. **SD** denotes "Standard deviation".

ABTS free radical scavenging activity

The ABTS free radical scavenging assay result by *L. speciosa* leaves extracts are shown in **Table 6. & Fig 3B**.

Table 6. ABTS free radical scavenging activity of *L. speciosa* leaves extracts.

**Conc.(µg/mL)**	**Extract**	**% of inhibitions**			**Mean ± SD**
		**1**	**2**	**3**	
	Methanol	11.42	11.57	12.62	11.87 ± 0.65
	Ethanol	10.06	10.6	11.2	10.62 ± 0.57
**20**	Acetone	9.32	9.38	9.89	9.53 ± 0.31
	n-Hexane	8.72	8.82	9.49	9.01 ± 0.42
	Ascorbic acid	14.68	14.87	15.51	15.02 ± 0.43
	Methanol	26.39	26.14	26.91	26.48 ± 0.39
**40**	Ethanol	25.16	25.68	26.5	25.78 ± 0.68
	Acetone	22.12	22.35	22.94	22.47 ± 0.42

	n-Hexane	26.04	26.17	27.02	26.41 ± 0.53
	Ascorbic acid	47.35	47.25	48.32	47.64 ± 0.59
	Methanol	60.55	61.17	61.49	61.07 ± 0.48
	Ethanol	54.91	55.16	55.77	55.28 ± 0.44
**80**	Acetone	47.79	48.06	48.9	48.25 ± 0.58
	n-Hexane	43.18	43.05	44.21	43.48 ± 0.64
	Ascorbic acid	73.67	73.55	73.76	73.66 ± 0.11
	Methanol	70.51	70.88	71.34	70.91 ± 0.42
	Ethanol	66.18	66.32	66.79	66.43 ± 0.32
**100**	Acetone	57.49	57.7	58.45	57.88 ± 0.50
	n-Hexane	49.44	49.84	50.09	49.79 ± 0.33
	Ascorbic acid	82.79	82.87	83.25	82.97 ± 0.25
	Methanol	83.08	83.25	84.02	83.45 ± 0.50
	Ethanol	76.58	76.65	76.99	76.74 ± 0.22
**150**	Acetone	68.64	68.62	68.84	68.7 ± 0.12
	n-Hexane	62.55	62.74	62.96	62.75 ± 0.21
	Ascorbic acid	92.51	93.02	93.86	93.13 ± 0.68

Note. **1, 2,** and **3** represent three independent experiments. **SD** denotes "Standard deviation".

Super oxide free radical scavenging activity

The Super oxide free radical scavenging assay results by *L. speciosa* leaves extracts are shown in **Table 7. & Fig 4A**.

Table 7. Super oxide radical scavenging activity of *L. speciosa* leaves extracts.

Conc.(µg/mL)	Extract	% of inhibitions			Mean ± SD
		**1**	**2**	**3**	
**20**	Methanol	17.81	17.66	18.59	18.02 ± 0.50

	Ethanol	11.57	12.31	12.78	12.22 ± 0.61
	Acetone	9.01	9.02	10.56	9.53 ± 0.89
	n-Hexane	11.51	11.36	11.81	11.56 ± 0.23
	Ascorbic acid	15.06	15.15	16.26	15.49 ± 0.67
**40**	Methanol	25.07	24.53	25.4	25 ± 0.44
	Ethanol	22.33	22.48	22.03	22.28 ± 0.23
	Acetone	18.18	18.89	19.27	18.78 ± 0.55
	n-Hexane	24.17	24.35	24.47	24.33 ± 0.15
	Ascorbic acid	35.33	36.02	36.68	36.01 ± 0.68
**80**	Methanol	52.12	51.48	53.39	52.33 ± 0.97
	Ethanol	42.14	42.48	42.79	42.47 ± 0.33
	Acetone	27.87	28.81	29.96	28.88 ± 1.05
	n-Hexane	38.37	38.89	39.38	38.88 ± 0.51
	Ascorbic acid	65.85	66.25	67.79	66.63 ± 1.02
**160**	Methanol	66.7	67.25	68.37	67.44 ± 0.85
	Ethanol	62.59	63.54	64.34	63.49 ± 0.88
	Acetone	43.97	44.46	44.92	44.45 ± 0.48
	n-Hexane	53.81	54.53	55.07	54.47 ± 0.63
	Ascorbic acid	85.51	86.19	87.08	86.26 ± 0.79
**300**	Methanol	80.71	81.02	81.9	81.21 ± 0.62
	Ethanol	76.65	77.26	77.78	77.23 ± 0.57
	Acetone	58.34	59.4	60.25	59.33 ± 0.96
	n-Hexane	64.72	65.3	66.24	65.42 ± 0.77
	Ascorbic acid	92.42	92.66	93.11	92.73 ± 0.35

Note. **1, 2,** and **3** represent three independent experiments. **SD** denotes "Standard deviation".

Nitric oxide radical scavenging activity

The result of the nitric oxide free radical scavenging assay by *L. speciosa* leaves extracts are shown in **Table 8. and Figure 4B**.

Table 8. Nitric oxide radical scavenging activity of *L. speciosa* leaves extracts.

Conc.(µg/mL)	Extract	% of inhibitions			Mean ± SD
		1	2	3	
**20**	Methanol	13.02	13.39	14.27	13.56 ± 0.64
	Ethanol	17.51	17.71	18.42	17.88 ± 0.48
	Acetone	9.65	9.66	9.67	9.66 ± 0.01
	n-Hexane	8.48	8.79	8.92	8.73 ± 0.23
	Ascorbic acid	19.04	18.72	20.41	19.39 ± 0.9
**40**	Methanol	22.61	23.14	24.6	23.45 ± 1.03
	Ethanol	27.06	27.05	27.1	27.07 ± 0.03
	Acetone	18.97	18.5	19.5	18.99 ± 0.50
	n-Hexane	20.27	21.41	21.98	21.22 ± 0.87
	Ascorbic acid	38.02	38.26	39.67	38.65 ± 0.89
**80**	Methanol	38.1	38.13	39.36	38.53 ± 0.72
	Ethanol	44.73	46.1	46.87	45.9 ± 1.08
	Acetone	36.3	37.43	37.84	37.19 ± 0.80
	n-Hexane	33.82	34.68	34.91	34.47 ± 0.56
	Ascorbic acid	58.04	59.81	60.89	59.58 ± 1.44
**160**	Methanol	58.01	58.08	58.27	58.12 ± 0.13
	Ethanol	67.05	67.24	67.7	67.33 ± 0.33
	Acetone	50.81	51.11	52.43	51.45 ± 0.86
	n-Hexane	42.22	43	43.93	43.05 ± 0.86
	Ascorbic acid	76.23	77.64	78.93	77.6 ± 1.35
**300**	Methanol	71.85	72.16	73.34	72.45 ± 0.79
	Ethanol	79.69	79.8	80.33	79.94 ± 0.34

Acetone	65.81	66.11	67.49	66.47 ± 0.90
n-Hexane	55.95	55.24	56.51	55.9 ± 0.64
Ascorbic acid	90.89	91	91.5	91.13 ± 0.33

Note. **1, 2,** and **3** represent three independent experiments. **SD** denotes "Standard deviation".

Brine shrimp lethality assay

The value of LC_{50} indicates the cytotoxic effect of *L. speciosa* leaf extracts. The concentration-response relationship was analyzed using a nonlinear regression curve fit, where the coefficient of determination, $R^2 = 0.9836$, yielded an LC_{50} value of 601.8 µg/mL (**Table 9. & Fig 5**).

Table 9. Concentration-dependent brine shrimp lethality assay of *L. speciosa* methanol extract.

Methanol extract of *L. speciosa* conc. (µg/mL)	Total shrimp	No. of nauplii dead in tubes				No. of nauplii Live	Mortality (%)
		1	2	3	Mean		
25	20	1	1	1	1	19	5
50	20	1	1	1	1	19	5
100	20	3	3	3	3	17	15
200	20	5	5	5	5	15	25
400	20	7	7	7	7	13	35
800	20	12	11	13	12	8	60

Note. **1, 2,** and **3** represent three independent tubes.

α-amylase assay

The α-amylase inhibitory activity of *L. speciosa* leaf extracts is summarized in **Table 10.** and illustrated in **Fig 6**. The extracts demonstrated dose-dependent inhibition of α-amylase activity, which was evaluated in comparison to the reference standard, Acarbose.

Table 10. α-amylase inhibitory activity of *L. speciosa* leaves extracts

Conc.(µg/mL)	Extract	% of inhibitions			Mean ± SD
		1	2	3	
50	Methanol	5.02	5.15	6.54	5.57 ± 0.84
	Ethanol	6.24	6.75	7.08	6.69 ± 0.42
	Acetone	7.1	7.41	8.05	7.52 ± 0.48
	n-Hexane	4.41	4.6	5.21	4.74 ± 0.42
	Acarbose	8.67	8.87	9.22	8.92 ± 0.28
100	Methanol	10.37	10.62	11.62	10.87 ± 0.66
	Ethanol	13.35	13.49	14.11	13.65 ± 0.40
	Acetone	11.8	12.14	12.84	12.26 ± 0.53
	n-Hexane	11.36	12.14	12.44	11.98 ± 0.56
	Acarbose	18.07	18.24	18.86	18.39 ± 0.42
200	Methanol	21.72	22.01	23.14	22.29 ± 0.75
	Ethanol	27.35	27.76	28.77	27.96 ± 0.73
	Acetone	20.36	20.87	21.47	20.9 ± 0.56
	n-Hexane	16.84	17.05	17.11	17 ± 0.14
	Acarbose	34.17	35.11	35.84	35.04 ± 0.84
400	Methanol	44.13	44.33	45.28	44.58 ± 0.61
	Ethanol	49.14	49.33	50.42	49.63 ± 0.69
	Acetone	38.48	39.27	40.12	39.29 ± 0.82
	n-Hexane	33.63	33.78	34.17	33.86 ± 0.28
	Acarbose	63.76	64.21	64.6	64.19 ± 0.42

	Methanol	55.07	56.15	56.81	56.01 ± 0.88
	Ethanol	60.41	61.13	61.52	61.02 ± 0.56
**500**	Acetone	45.25	45.81	46.04	45.7 ± 0.41
	n-Hexane	40.74	40.91	41.23	40.96 ± 0.25
	Acarbose	78.55	78.7	79.3	78.85 ± 0.40

Note. **1, 2,** and **3** represent three independent experiments. **SD** denotes "Standard deviation".

α-glucosidase assay

The α-glucosidase inhibitory activity of *L. speciosa* leaf extracts is summarized in **Table 11** and illustrated in **Fig 6**. The extracts demonstrated dose-dependent inhibition of α-glucosidase activity, which was evaluated in comparison to the reference standard, Acarbose.

Table 11. α-glucosidase inhibitory activity of *L. speciosa* leaves extracts

Conc.(μg/mL)	Extract	% of inhibitions			Mean ± SD
		1	2	3	
	Methanol	16.46	14.77	15.75	15.66 ± 0.85
	Ethanol	11.87	12.1	14.67	12.88 ± 1.55
**50**	Acetone	9.9	10.54	11.15	10.53 ± 0.63
	n-Hexane	12.86	13.05	15.67	13.86 ± 1.57
	Acarbose	21.17	20.51	22.22	21.3 ± 0.86
	Methanol	21.16	23.24	20.01	21.47 ± 1.64
	Ethanol	19.25	18.83	20.84	19.64 ± 1.06
**100**	Acetone	18.16	19.46	17.55	18.39 ± 0.98
	n-Hexane	21.5	22.99	22.68	22.39 ± 0.79
	Acarbose	35.04	34.1	36.73	35.29 ± 1.33
	Methanol	47.36	47.86	46.2	47.14 ± 0.85
**200**	Ethanol	43.71	44.97	44.37	44.35 ± 0.63
	Acetone	34.75	32.63	33.39	33.59 ± 1.07

	n-Hexane	29.09	28.03	27.81	28.31 ± 0.68
	Acarbose	57.95	55.02	55.93	56.3 ± 1.5
**400**	Methanol	61.07	60.34	62.25	61.22 ± 0.96
	Ethanol	55.03	55.12	57.43	55.86 ± 1.36
	Acetone	45.91	48.12	45.02	46.35 ± 1.6
	n-Hexane	35.05	34.38	36.32	35.25 ± 0.99
	Acarbose	70.02	71.55	70.23	70.6 ± 0.83
**500**	Methanol	74.16	75.33	73.29	74.26 ± 1.02
	Ethanol	60.17	60.69	62.8	61.22 ± 1.39
	Acetone	51.05	50.8	52.29	51.38 ± 0.8
	n-Hexane	40.06	42.44	41.28	41.26 ± 1.19
	Acarbose	86.62	85.15	85.54	85.77 ± 0.76

Note. **1, 2,** and **3** represent three independent experiments. **SD** denotes "Standard deviation".
